# Recent Advances in Enhancement Strategies for Electrochemical ELISA-Based Immunoassays for Cancer Biomarker Detection

**DOI:** 10.3390/s18072010

**Published:** 2018-06-22

**Authors:** Sunil K. Arya, Pedro Estrela

**Affiliations:** Centre for Biosensors, Bioelectronics and Biodevices (C3Bio) and Department of Electronic & Electrical Engineering, University of Bath, Claverton Down, Bath BA2 7AY, UK

**Keywords:** electrochemical ELISA, serum, immunoassay, antibodies, cancer detection

## Abstract

Electrochemical enzyme-linked immunosorbent assay (ELISA)-based immunoassays for cancer biomarker detection have recently attracted much interest owing to their higher sensitivity, amplification of signal, ease of handling, potential for automation and combination with miniaturized analytical systems, low cost and comparative simplicity for mass production. Their developments have considerably improved the sensitivity required for detection of low concentrations of cancer biomarkers present in bodily fluids in the early stages of the disease. Recently, various attempts have been made in their development and several methods and processes have been described for their development, amplification strategies and testing. The present review mainly focuses on the development of ELISA-based electrochemical immunosensors that may be utilized for cancer diagnosis, prognosis and therapy monitoring. Various fabrication methods and signal enhancement strategies utilized during the last few years for the development of ELISA-based electrochemical immunosensors are described.

## 1. Introduction

Cancer is one of the major causes of mortality in the world. Many factors, including exposure to cancer-causing reagents, exposure to radiation, infections, genetic modifications, etc., can disrupt the cells and result in their modification and proliferation causing the generation of cancer in different parts of the body. Its diagnosis based on visual symptoms is not recommended as such symptoms appear in later stages of cancer, when there are no efficient therapies. Thus, it is advised to diagnose it in early stages, when useful treatment is possible, in order to achieve longer survival of cancer patients [1]. To achieve early stage diagnosis researchers have proposed the use of proteins and oligonucleotides released in the body during the early stages of cancer and not present in the same concentrations in healthy individuals. Such molecules are known as biomarkers and different types of cancers release different biomarkers, whose detection and estimation can provide very valuable information regarding cancer type and its stage. Thus, it is very important to develop systems, which are simple, low cost and can provide sensitive and specific estimation of such biomarkers [2]. Further, taking into account population and cancer stage variability as well as low levels of biomarkers in early stages in cancer, it is recommended to identify and test panels of multiple biomarkers for better accuracy in diagnosis. Also, it is desired to detect these biomarkers in a non-invasive or minimally invasive manner with high selectivity, sensitively and free from false positives and false negatives. Commonly employed methods of cancer detection such as enzyme-linked immunosorbent assay (ELISA), western blotting, optical, electrochemical, fluorescence or radio immunosensor-based systems also utilize biomarkers for analysis and their estimated levels are related to cancer stage and inform cancer therapy [3,4]. With advances in cancer biology and immunology, researchers have discovered various potential biomarkers specific to particular cancers and related to the bio-mechanism of cancer cells.

Till date, mainly optical sandwich ELISA-based detection of biomolecules is employed in clinical practice and commonly considered as the gold standard method. These assays use antibodies for specific identification and quantification of the desired antigen/biomarker in a process known as immunoassay; sensors used for these assays are known as immunosensors [5,6]. In the medical diagnostics industry, traditional optical ELISA is usually carried out in 96 well plates. Suppliers provide kits of reagents and 96 well plates for desired analytes testing and estimation. In such kits, 96 well plates generally come with a primary antibody coated into the wells of the plate via physical adsorption followed by blocking to prevent non-specific binding. The kits also provide operating procedures. In brief, an antigen sample is first incubated with primary antibodies in the well for the required time to make antibody–antigen complex. After incubation, plate is usually washed with wash buffer provided by the kit provider. After washing antigen-antibody complex is incubated with enzyme tagged detection antibody to form antibody-antigen-antibody sandwich. After incubating for the desired time, followed by washing with wash buffer, the complex is incubated with enzyme substrate and indicator dye. During incubation, the enzymatic reaction results in change of color for indicator dye, which on measurement using optical reader provide the absorbance value. Absorbance value on comparison with standard solution calibration provide the analyte concentration. The whole testing procedure is quite lengthy and often requires an expensive optical reader for analyte estimation. However, the use of a sandwich method provides amplified response and thus results in better detection range. In brief, optical ELISA provides highly reproducible, sensitive and specific, quantitative data that makes it an advantageous biotechnological tool in scientific research and clinical diagnosis. However, optical ELISA suffers from tedious/laborious procedures, necessity for centralized laboratory equipment, and a relatively high sample volume is required. Moreover, the detection limit of conventional ELISA is barely less than the nanomolar concentration level, which is inadequate to reach the clinical threshold of many protein biomarkers, especially in the early stage of diseases.

Electrochemical assays have shown the promise to overcome these issues. Electrochemical assay provides the advantage of easy procedure, portable instrumentation, low volume and faster measurements. However, like for optical ELISA in 96 wells performing large multiplexing simultaneously, not much success has been reported in electrochemical assays. Among electrochemical assays, electrochemical ELISA has shown promise, as it combines the advantages of optical ELISA like sensitive and specific, multiplexing, quantitative data with advantages of an electrochemical assay like faster, lower sample volume, low cost instrumentation, etc. Thus, to shorten the time required and to improve the response and characteristics of traditional optical ELISA, various researchers have proposed newer technologies via development of improved sensor surfaces and detection probes. Also, to reduce cost, easier testing and shorter measurement time, sandwich-based electrochemical ELISAs have been proposed, which utilize the specificity of optical ELISA and advantages of electrochemical measurements to achieve better response and characteristics for desired analyte estimation. In contrast to optical ELISA, electrochemical ELISA uses a potentiostat/galvanostat for signal measurement in research laboratories. Though at present there are not many commercial electrochemical ELISA based immunosensors, the required instrumentation is available and as such there is huge potential for such sensors and their commercialization. Furthermore, the ease of miniaturization of required electronics has potential for smaller, simpler and low cost systems for such measurement. In brief, it has been suggested that, to overcome limitations of optical ELISA, whilst maintaining the advantages of traditional assays, electrochemical immunosensors may provide a workable alternative [7,8,9]. Electrochemical immunosensors utilizing potential, current or impedance based techniques may provide the desired sensitivity in very low volume samples at faster rate of analysis along with ease of fabrication, measurement and mass production at low cost [9,10,11,12]. Keeping these advantages in mind, researchers have recently focused on the development of electrochemical ELISA-based systems to combine the advantages of sandwich assays used in optical ELISA and electrochemical detection [13]. Electrochemical ELISA enjoys the specificity and signal amplification obtained by the use of synchronized binding of the recognition molecule and detection molecule, along with high sensitivity, low detection limit, easy handling and easy detection in miniaturized format provided by electrochemical detection. With evolving material and surface chemistries along with advancing bio- and nano-technologies, electrochemical ELISA based immunosensors have been gaining much interest and promising to replace traditionally used optical ELISA to achieve faster, more sensitive, cheaper and reliable detection of cancer biomarkers in for early stage diagnosis [14,15]. The present review describes various new ways reported by researchers in the last 3 to 4 years for developing and improving sandwich-based electrochemical ELISA for cancer biomarker detection. Authors in many of these reports validated their approaches in spiked/real samples in vitro. There is no information regarding commercialization of any of these sensors at present, however these reports may pave way for better and faster diagnostic of cancer at earlier stages in the near future. Also, there are various useful reviews that have been published for electrochemical immunosensor-based cancer biomarker detection in past using nanoelectrodes, arrays and microfluidics [16,17,18]. Thus, in the future, combining the new advancements in sensor surfaces and detection probes described here with nanoelectrode arrays or microfluidics will further enhance the chances of achieving better sensitivity and detection limits required for early stage measurements of biomarkers. 

### 1.1. Electrochemical Sandwich ELISA

Electrochemical sandwich ELISA is a branch of electrochemical immunoassays where the recognition of a desired target is done using a traditional sandwich assay and detection is achieved using an electrochemical method [19,20,21]. These immunoassays mainly involve three layers: immobilized biorecognition molecule (probe), target analyte that binds specifically to the biorecognition molecule, followed by binding of a secondary recognition molecule with an electrochemically active signal tag. For signal measuring, the electrochemical signal tag either provides the signal directly or a reaction with a substrate is induced afterwards [22,23]. The generated signal is directly proportional to the analyte concentration. This type of sensing involving sandwiching of target analyte between two highly specific capturing and recognition molecules, provides a high level of sensitivity and specificity and makes it suitable for early stage detection of cancer biomarkers [14]. For capturing and recognition molecules one can utilize combinations of suitable molecules ranging from antibodies, aptamers, DNA base sequences, bacteriophages, peptide nucleic acid sequences, etc. [2]. And for sensitive detection researchers have utilized various tags involving redox enzymes, metallic particles, quantum dots, etc., which they have used directly or in combination with another matrix for enhanced loading [6,14,24,25]. Other than these, the activity and recognition ability of developed sensors also depend on how and where the capturing molecule is immobilized and how well it can interact with the target analyte. Thus, innovative methods for binding of capturing agents on the desired surface (hereafter referred to as matrix, to reflect the modification of the electrode with different molecular and polymeric layers) for sensitive analyte capture are required for developing novel immunosensors. Further, the selection and development of a suitable matrix for binding of capturing agent is crucial to achieve optimum response from the assay [5]. Thus, for development of novel electrochemical ELISA-based immunosensors, research groups are working on finding newer and better matrices along with novel methods of binding capturing agents on the desired matrices and have proposed numerous approaches for higher binding of capturing molecules with better retained activity. Researchers have also proposed new and innovative methodologies to amplify the signal generated by binding events of target and recognition molecule. This review will focus mainly on the recent advances made by various groups in methods of making such electrochemical ELISA-based immunosensors for cancer biomarkers detection using innovative surface chemistries and materials along with their measuring methodology and response to analyte. For development of enhancement strategies, researchers have utilized various biomarkers such as CEA, AFP, PSA HER2, SCC, CA 125, CA 19-9, etc. Among these biomarkers CEA, AFP and PSA have gained much attention as model biomarkers for developing enhancement strategies. In a normal person, the cut-off concentration for CEA, AFP, CA 125 and PSA are found to be 3 ng/mL, 10 ng/mL, 46 U/mL and 4 ng/mL, respectively, and higher concentrations are oftern related to cancer stages. Immunosensors and enhancement strategies are normally investigated keeping these ranges in mind; the immunosensor is useful if it can detect concentrations lower than the cut off limit.

### 1.2. General Mechanism of Enhancement Strategies

**Figure 1:**
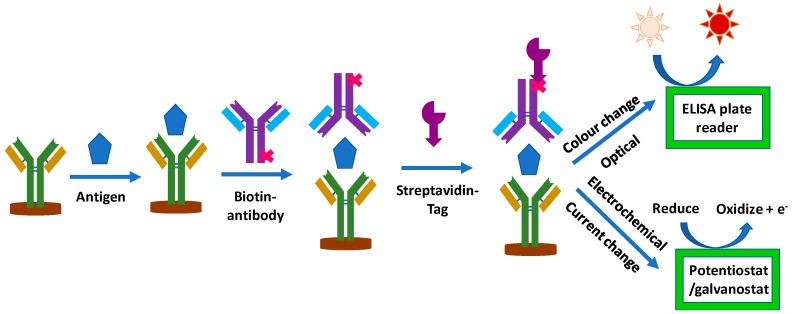
General schematic for immunosensor functioning and detection.

In sandwich-based electrochemical ELISA, signals can be enhanced by increasing the capturing efficiency via the use of better antibodies or by their higher loading on sensor surface. Signals can also be enhanced via detection probes containing a larger number of detection tags. Thus, sensor surfaces where capture antibodies are immobilized and detection probes containing detection antibodies and tags play the main roles in achieving enhancement in signal. In general, enhancement in response has been achieved using modified sensor surfaces or improved detection probes. Using modified surfaces, researchers have tried to increase the surface area via use of nanomaterials and their composites, thus resulting in higher loading of antibodies. Also attempts were made to immobilize capturing antibodies in the desired orientation for enhance capturing efficiency, thus resulting in signal enhancement. In use of improved detection probes, researchers have utilized high surface area of nanomaterials and composites for loading of larger number of detection antibodies with tags or catalytic materials. On interaction of detection probe with antibody-antigen complex, one of the detection probe binds to antigen but many more are also available for further catalytic reaction of the detection substrate. In case of enzymatic tags, a larger number of tags results in larger conversion of specific analyte, thus resulting in larger quantity of detection molecules. Also, in the case of nanomaterial based catalytic tags on detection probe, large number of tags result in larger catalytic conversion of target substrate, thus resulting in higher response. Figure 1 shows the general detection strategy for optical and electrochemical ELISA.

## 2. Matrix Selection, Modification and Development of Immunosensors

The development and properly working immunosensors involves the selection and preparation of a binding matrix followed by immobilization of capturing molecule on the surface of the electrode. The matrix for an electrochemical sensor can comprise monolayers, polymers, carbon based materials, nanomaterials or their composites [13,14,25,26]. Most commonly utilized capturing molecules include antibodies, antibody fragments, DNA/RNA aptamers and peptide aptamers, which are immobilized directly on a conducting/semiconducting electrode surface or on a pre-modified electrode surface via physical, entrapment or covalent methods [27]. Other than these, researchers have also employed oriented biomolecular immobilization approaches either by using engineered capturing molecules, or by using molecules such as protein A, which allows the binding of antibodies in an ‘upright’ position for best activity. In any type of chosen method for binding of capturing molecule, the main emphasis during immobilization is to retain or enhance the capturing molecule activity and stability. Further, binding can also be characterized based on the chosen matrix [27].

In recent years, carbon-based matrices involving graphene oxide (GO) [28], reduced graphene oxide (rGO) [29], graphene sheets [30,31,32], carbon nanotubes [24,33] and their composites with nanoparticles [33,34,35], polymers [36], etc., have recently attracted much attention owing to their high conductivity, large surface area, and stability. In most of the cases, blocking of free surface areas on the sensor chip after capture molecule binding was achieved using BSA solution incubation.

In one example, Gao et al. utilized graphite to prepare GO, which was then reduced and nitrogen doped before coating on a glassy carbon electrode (GCE) for antibody binding via glutaraldehyde chemistry [28]. They further showed the use of β-cyclodextrin-graphene (β-CD-GR) for GCE coating and binding of capture antibody (Ab1)-adamantine (ADA) via physical adsorption [37]. A few other researchers have also utilized the CD-GS based matrix for immunosensor development [38,39]. Li et al. described another approach where single walled carbon nanotubes (SWCNTs) were mixed with l-cysteine modified chitosan (CS) to obtained thiol terminated CSSH-SWCNTs, which can be immobilized on gold surfaces from one side and can be employed for gold nanoparticles (AuNPs) binding on other side for larger surface area and Ab1 binding [40]. Similar to this, Wang et al. reported the use of AuNPs decorated mercapto-functionalized graphene sheets (Au@SH-GS) as matrix on GCE for Ab1 binding [41]. 

**Figure 2:**
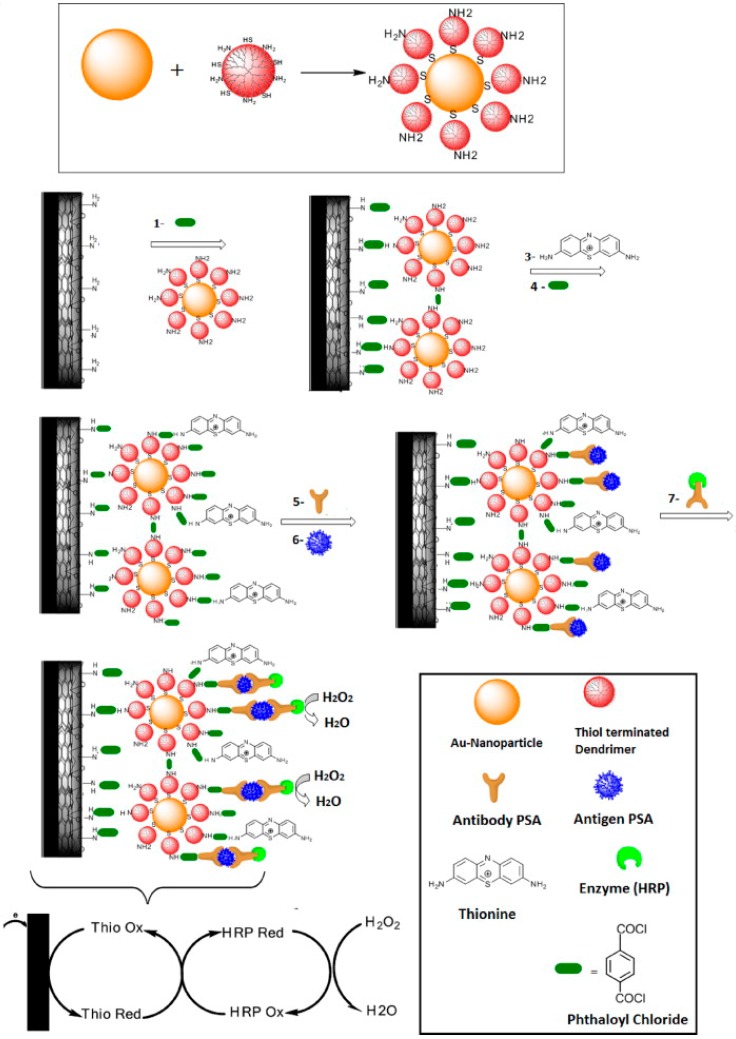
Illustration of the stepwise process for PSA immunosensor fabrication. Briefly, to prepare sensor electrode, physical mixture of MWCNTs and IL was coated onto GCE which was then modified with PAMAM decorated gold nanoparticles via phthaloyl chloride chemistry. Phthaloyl chloride chemistry was further utilized for thionine and anti-PSA immobilization. Reproduced with permission from [42].

In composites with polymers, Feng et al. described use of hierarchically aloe-like gold microstructures (HAG)/polyaniline (PANI)/rGO, where PANI and HAG were electrochemically deposited on rGO coated GCE [36]. In other study, Kavosi et al. described the development of immunosensor using gold nanoparticles/polyamidoamine dendrimers (AuNPs/PAMAM dendrimer) loaded MWCNTS/CH/ionic liquid (IL) nanocomposite onto GCE surface [42]. Figure 2 shows the schematic for the sensor development and detection procedure. AuNPs-IL-rGO nanocomposite has also been utilized for immunosensor development [43,44]. Other than these, many other composites like gold-(3-aminopropyl)triethoxysilane-GS (Au@APTES-GS) [45], Au-GR [46], AuNPs/thionine(Thi)-CNTs [47], etc. have been utilized and shown to provide novel matrices for immunosensor development.

Other than graphene or CNTs, gold has been used as matrix independently or with many other matrices in composite form for biofunctionalization. Use of such nano and hybrid materials, whose properties can be controlled and tailored in a desired manner have provided a viable opportunity to develop clinically relevant immunoassays in the biomedical arena. Some examples include use of electrochemically deposited AuNPs [48,49,50,51], AuNPs-Chitosan [52,53,54], nanoporous gold (NPG) prepared by acid based removal of silver from silver gold alloy [55], MoS_2_-Au hybrids [56], Au-multifunctional mesoporous silica (MCM-41) [57], poly(*o*-phenylenediamine)-AuNPs [58], etc.

Other than these nanomaterials and composites, other materials such as polymers, magnetic materials, electrodeposited films and monolayers has also been utilized for immunosensor development. Wang et al. described the use of Ab1 tagged Dynabeads conjugates [59], while Zhou et al. described CD coated GCE electrode for immunosensor development [60]. Further, PAMAM modified GCE [61], cysteine monolayer on gold [62], etc. have also been utilized for advanced immunosensor development. Table 1 summarizes the various approaches used for immunosensor development.

## 3. Electrochemical ELISA Based Detection

For signal detection in electrochemical ELISA-based sensors, researchers have explored the use of various electrochemical amperometric and voltammetric techniques including differential pulse voltammetry (DPV), linear sweep voltammetry (LSV), stripping voltammetry and square-wave voltammetry (SWV). In general, once the analyte is captured, the sensor is incubated with the detection molecule tagged with an electroactive agent, such as a redox molecule, nanoparticle, and quantum dot, etc., or with an enzyme capable of generating electroactive species for signal measurement [17]. Use of redox enzyme-based indicator systems is most common and wildly applied in electrochemical ELISA-based immunosensors [105]. Traditionally a 1:1 ratio of redox enzyme and detection molecule is used for amplification and signal measurement. With the advances in material science and chemistry newer nano and hybrid materials have been explored in recent years to enhance the amplification of the signal. These advanced materials act as carriers for loading of multiple enzyme molecules and thus enhance the signal [10,24,25]. However, the use of redox tags and enzyme-mimicking molecules are also receiving much attention in the development of advanced immunosensors. The use of nano labels have also provided the opportunity to achieve better signals and to develop better immunosensors [106]. The signal is usually measured via an amperometric or voltammetric technique using a potentiostat or other low cost electrochemical systems, mainly in a three-electrode configuration. The following sections have been divided in a way to provide more details of different strategies employed for enhanced signal detection. Table 2 shows the details of various strategies used for the development of detection probes for enhanced detection. Furthermore, Table 3 shows the various characteristics of the developed immunoassays using electrochemical ELISA.

### 3.1. Redox Enzyme Based Detection

The majority of immunosensors till date use redox enzymes for signal amplification and to enhance sensitivity of the immunoassay [22]. In such assays, the detection molecule, which binds to the antigen at a second binding site, is either tagged directly to a redox enzyme or is labeled with a tag capable of binding a modified redox enzyme. After redox enzyme binding, the enzyme catalyzes its substrate and generate an electroactive product, whose measurement give the information regarding the target analyte. Redox enzymes are used either as free molecules or after loading them on metallic or carbon-based nanomaterials for higher signal. 

#### 3.1.1. Free Redox Enzyme and Redox Enzyme with Nanomaterial Based Enhancement

**Figure 3:**
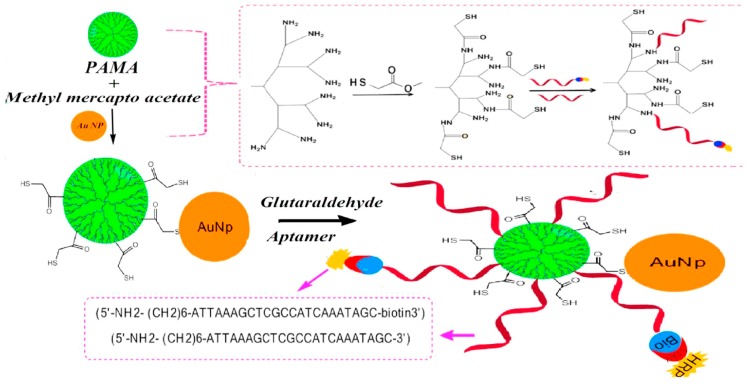
Illustration of the stepwise process for PSA immunosensor fabrication. Reproduced with permission from [70].

In a free redox enzyme and redox enzyme with nanomaterial approach, redox enzyme tagged with detection antibody catalyzes its substrate to generate the electrochemical response. This section describes the various approaches investigated by researchers for loading of redox enzymes onto various nanomaterials for enhancing their concentration during the immunoassay, which in turn increases the signal response. Among various nanomaterials, gold nanoparticles have been investigated most. They have also been used in combination with CNTs and other composites. In the last few years, the use of free enzymes for enhancement strategy is rarely utilized as more advanced strategies have been developed. In one such study of free enzyme based system, Patris et al. utilized the horseradish peroxidase (HRP) tagged detection antibody (Ab2) for signal detection in HER2 immunosensor. For signal enhancement, the antibody-antigen complex was incubated with detection probe for 20 min followed by testing in citrate buffer containing 2.5 mM hydrogen peroxide (H_2_O_2_) and the reduction current for added hydroquinone (HQ) was monitored at −280 mV. Results showed detection of HER2 at two concentrations: 1 and 200 µg/mL [63]. For better enhancement, researchers have developed many nanomaterial-tagged redox enzymes based strategies and achieved improved detection limit and sensitivity. In one such study, Feng et al. utilized physically loaded Ab2 and HRP onto AuNPs-PANI@CNTs nanocomposites for signal enhancement in their immunosensor for CEA detection [47]. Detection probe was developed by chemical reduction of aniline in CNT presence followed by electrostatic assembly of AuNPs. During immunoassay, Ab1-antigen complex is incubated with detection probe for 55 min at 37 °C and CEA detection down to 0.008 ng/mL via DPV was achieved in phosphate buffer saline (PBS) containing 4 mM H_2_O_2_. They observed two linear ranges, and ascribed the lower concentration range to isadsorption-controlled processes on the electrode, whereas linearity in higher concentrations was attributed to diffusion controlled processes on the electrode. In other study, Kayosi et al. described the use of HRP-prostate specific antigen (PSA) aptamer-modified AuNP-PAMAM conjugate for signal enhancement [70]. They utilized glutaraldehyde chemistry for immobilizing PSA aptamer and HRP-PSA aptamer (prepared using streptavidin-biotin coupling) onto detection probe. With this probe they achieved PSA detection down to 10 fg/mL when tested by DPV scanning. Figure 3 shows the schematic for detection probe development. Further, AuNPs have been utilized by Zhang et al. in the development of AuNPs modified SBA-15 (Au@SBA-15) based detection probe for carbohydrate antigen 19-9 (CA 19-9) estimation [74]. Ab2-HRP was conjugated onto Au@SBA-15 via Au–NH^3+^ or Au–SH affinity and enhanced direct electron transfer (DET) was utilized for signal enhancement. With such probe they were able to achieve CA 19-9 detection down to 0.01 U/mL.

**Figure 4:**
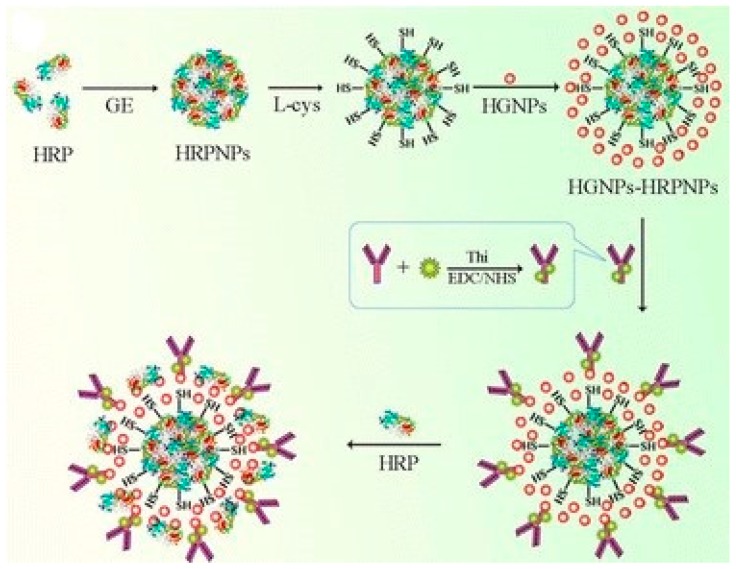
Synthesis process of hollow Au-NPs-HRP-NPs and preparation procedure of HRP-hollow Au-NPs-HRP-NPs-Thi@Ab2 bioconjugate. Reproduced with permission from [40].

The use of HRP modified hollow AuNPs has been described by Li et al. for the development of an AFP immunosensor. In their method, hollow AuNPs were synthesized by HAuCl_4_ reduction in N_2_ environment using sodium borohydride (NaBH_4_), sodium citrate and CoCl_2_∙6H_2_O mixed solution and then modified with l-cysteine modified HRP-NPs. Physically immobilized Thi-anti-AFP based probe then achieved AFP detection down to 8.3 pg/mL when incubated with Ab1-antigen for 30 min at room temperature and the DPV signal was recorded in the presence of H_2_O_2_ [40]. Figure 4 shows the schematic for HRP-HRP-NPs-hollow AuNPs-Thi@anti-AFP bioconjugates development. Moving away from gold, Wang et al. described the use of Fe_3_O_4_ and HRP modified mesoporous silica nanoparticles (MSNs) based detection probe for an AFP immunosensor. The detection probe with Ab2 and HRP were immobilized using glutaraldehyde chemistry, and showed AFP detection down to 4 pg/mL when tested via CV in PBS with and without 5 mmol/L H_2_O_2_ [64]. In another study, Wang et al. described silver nanoparticles (AgNPs) based detection probe for CEA estimation. For probe development anti-CEA and glucose oxidase (GOD) were physically immobilized onto Ag nanospheres prepared via an ethylene glycol (EG) and poly(vinyl pyrrolidone) (PVP) assisted method. For immunoassay, probe was incubated with Ab1-antigen for 1 h at 4 °C and the resulting complex was tested via DPV. With such probe they achieved CEA detection down to 0.27 pg/mL [56]. Further, Zhou et al. utilized HRP tagged BSA-nanosilver microspheres (Ag@BSA) to quantify CEA. Using enzymatic precipitation and amplification of tyramine signal, they achieved CEA detection down to 5.0 pg/mL via DPV method [108]. Tang et al. utilized HRP and Ab2 modified magnetic nanoparticles (MNPs) based strategy for PSA, PSMA, IL-6, and PF-4 estimation in ab array format. HRP and Ab2 were tagged onto MNP via biotin–streptavidin chemistry. Using HRP tag and added HQ as mediator they detected the target via DPV in the presence of H_2_O_2_ and achieved detection down to pg/mL range for all four analytes [81]. In other study, Uludag et al. utilized HRP and anti-PSA tagged AuNPs as detection probe TMB as mediator to achieve detection down to 0.2 ng/mL when tested via amperometric at −0.1 V in the presence of H_2_O_2_ [82].

#### 3.1.2. Redox Enzyme with Carbon Material Based Enhancement

To enhance the response of immunosensors in electrochemical ELISA, various carbon-based nanomaterials have been explored to increase the loading of redox enzyme tagged detection antibody probes. This section describes various carbon-based material investigated by researchers to enhance the sensitivity of immunosensor. Among various carbon-based nanomaterial, graphene oxide and reduced graphene oxide have gained maximum attention in recent years. Other than graphene, various carbon-based materials such as CNTs, MWCNTS, nanodots and nanocomposites, etc. have also been utilized for developing detection probes to achieve high sensitivity. Huang et al. described a Ag/Au NPs-graphene based enhancement strategy for CEA immunosensor: a 1,5-diaminonaphthalene (DN)-based Ag/Au–DN–GR probe was prepared simply by mixing Ag/Au (prepared via reduction) with DN-GR. The immunoassay with physically adsorbed anti-CEA onto Ag/Au–DN–GR showed CEA detection down to 8 pg/mL when incubated with Ab1-antigen for 40 min [34]. Similarly, a Au@Pd-GR composite was used by Yang et al. for detection probe development by immobilizing Thi, HRP and anti-CA19-9. It was observed that synergy between Au@Pd-GR and HRP resulted in three times higher response in the presence of H_2_O_2_ and the sensor exhibited CA19-9 detection down to 0.006 U/mL [72]. 

GR-PAMAM dendrimer conjugate based detection probe development was described by Shen et al. GR and PAMAM were conjugated via EDC/NHS chemistry and then utilized for anti-AFP and HRP binding using glutaraldehyde cross-linking. With this simple probe and hydroquinone as detection molecule, they achieved amperometric detection of AFP down to 0.45 ng/mL [50]. In other study, Yang et al. described the development of duel enzyme bio-catalyzed precipitation of 4-CN based immunosensor for α-fetoprotein (AFP) detection. For probe, HRP, GOD and anti-AFP were immobilized via EDC/NHS chemistry on carboxylated SWCNHs. With their probe they achieved AFP detection down to 0.33 pg/mL [73].

### 3.2. Redox Marker Based Detection

Other than redox enzymes, researchers have employed the use of redox active tags such as nanoparticles, quantum dots or organic/inorganic molecules for measuring signal from sandwich immunoassay. Such molecules are also either tagged directly to detection molecule in free form or after loading to other nanomaterials. Such tags on electrochemical oxidation/reduction provide the information of tags concentration which in turn can be related to the analyte concentration in the immunoassay.

#### 3.2.1. Free Redox Marker and Redox Marker with Metallic Nanomaterial Based Enhancement

**Figure 5:**
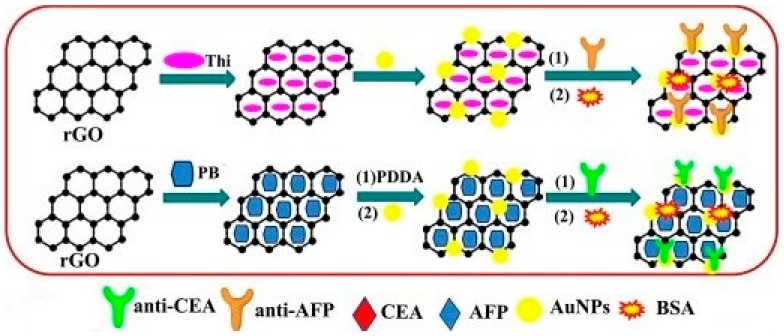
Preparation process of immunosensing probes. In brief, reduced GO was first treated with Thi/Pb for their electrostatic binding, which was then utilized for physical adsorption of pre-synthesized AuNPs onto Thi/rGO or PDDA modified PB/rGO. The bound AuNPs were then utilized for physical adsorption of desired antibodies. Reproduced with permission from [36].

This section describes various different types of redox markers either in free form or loaded onto nanomaterials, investigated by researchers to enhance the sensitivity of sandwich-based immunoassays. The higher the presence of redox markers suggests higher responses, thus loading of such markers onto nanomaterials has shown promise in enhancing the sensitivity of immunoassays. Among various nanomaterials, AuNPs have gained maximum attention for achieving higher loading of redox tags. Yang et al. described the use of 6-ferrocenyl hexanethiol tagged AuNPs based probe development for PSA detection. With high physical loading of anti-PSA onto Fc tagged AuNPs, they were able to detect PSA down to 5.4 pg/mL [68]. Lin et al. introduced the use of AuNPs-mesoporous carbon form (MCF) as redox tag. In immunoassay physically tagged anti-CEA on Au/MCF was incubated with Ab1-antigen for 40 min and attached Au/MCF tags were then utilized for silver-deposition by incubating with enhancer solutions in dark for 4 min at 37 °C. Results of CEA detection using anodic stripping analysis revealed detection down to 0.024 pg/mL [71]. Chitosan-AuNP based detection probe was described by Chen and Ma. They utilized CHIT-PB-AuNP and CHIT-FC-AuNP probes for CEA and AFP detection, respectively. Corresponding Ab2 were physically immobilized on desired conjugate and used for immunoassay. For measurement, detection probe mixture was incubated with Ab1-antigen for 45 min at 37 °C and DPV signal was recorded in PBS. With this scheme, they were able to detect AFP and CEA down to 0.03 ng/mL and 0.02 ng/mL, respectively [52]. In another study, Feng et al. described the development of anti-AFP_2,2_-AuNPs-Thi@rGO and anti-CEA_2,1_-AuNPs-PB@rGO bioconjugates as detection probe. Probes were easily prepared by mixing and physical adsorption. For measurement, detection probes with physically adsorbed Ab2 were incubated with Ab1-antigen for 50 min at 37 °C and DPV measurements were carried out in PBS. Results indicate that with their probes, CEA and AFP can be estimated simultaneously down to 0.12 ng/mL and 0.08 ng/mL, respectively [36]. Figure 5 shows the preparation process of immunosensing probes. Further, Liu and Ma described PB–CS-Au and Cd–CS-Au based immunoprobes for CEA and AFP detection. With physically adsorbed Ab2, they were able to detect CEA and AFP simultaneously down to 0.006 ng/mL for AFP and 0.01 ng/mL for CEA [44]. Cd^2+^ modified nanoporous TiO_2_ has also been utilized for probe development for carbohydrate antigen 15-3 (CA15-3) detection down to 0.008 U/mL [32].

**Figure 6:**
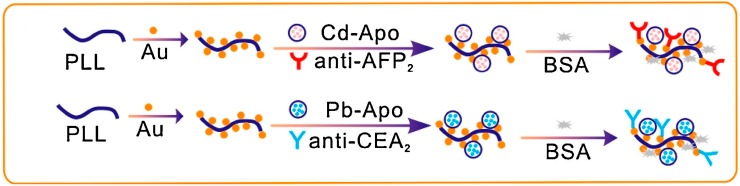
Preparation process of PLL-Au-Cd-Apo-Ab2 and PLL-Au-Pb-Apo-Ab2 signal tags. Reproduced with permission from [59].

In a different strategy, polymer-nanotags based signal probes were described by Wang et al. for AFP and CEA detection. Probes were prepared by mixing metal ions (Cd^2+^, Pb^2+^) modified Apo solution with PLL-Au nanocomposites, which was then utilized for physical adsorption of Ab2. For detection via SWV, they utilized captured metal ions during immunoassay to deposit bismuth film at −1.2 V and estimated AFP and CEA at −0.78 V and −0.53 V, simultaneously. With this scheme they achieved detection down to 4 pg/mL for both targets [59]. Figure 6 shows the preparation process of immunosensing probes. Wang et al. also described the use of PtPNPs-Cd^2+^ and PtPNPs-Cu^2+^ hybrids based detection probes for immunosensing. Cd^2+^ or Cu^2+^ ions modified PTPNPs were utilized for physical immobilization of Ab2 and DPV signals were recorded for CEA and AFP at −0.736 V and 0.004 V, respectively. Using these probes and incubation for 1 h at 37 °C with Ab1-antigen conjugate, they achieved detection down to 0.002 ng/mL and 0.05 ng/mL for CEA and AFP, respectively [29]. Similarly, Wang et al. described the use of nanocubes of copper and cadmium hexacyanocobaltate based probes for CEA and AFP immunosensing and achieved detection down to 0.0175 ng/mL and 0.0109 ng/mL for CEA and AFP respectively [53].

**Figure 7:**
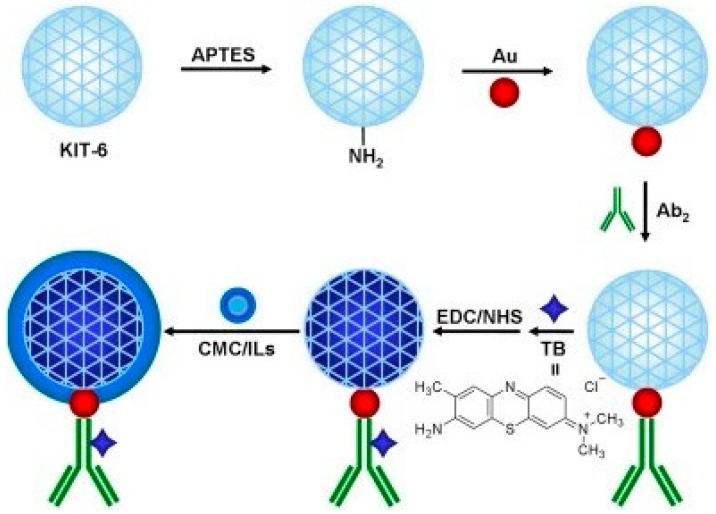
Preparation procedure of the TB/Au@KIT-6/CMC/ILs-Ab2 labels. Reproduced with permission from [45].

Use of AuNPs modified mesoporous carbon CMK-3 has been described by Wu et al. to develop detection probes for CEA and SCCA estimation, simultaneously. For detection probe Au@CMK-3-anti-CEA-neutral red and Au@CMK-3-anti-SCCA-thionine conjugate were prepared via EDC/NHS chemistry and assay results using these probes showed detection down to 0.013 ng/mL and 0.010 ng/mL for CEA and SCCA, respectively [66]. Cu^2+^ and Pb^2+^ tagged AuNPs have also been used by Xu et al. for CEA and AFP detection down to 4.6 pg/mL and 3.1 pg/mL, respectively [54]. In other approach, Wang et al. described AuNPs modified mesoporous silica KIT-6 (Au@KIT-6) as surface to bind Ab2 (anti-CEA) and toluidine blue (TB) mediator based strategy for immunosensor development for CEA detection. With the developed probe (TB/Au@KIT-6/CMC/ILs-Ab2), they incubated Ab1-antigen complex for 1 h and achieved detection of CEA down to 3.3  fg/mL [45]. Figure 7 shows the preparation process of the immunosensing probes. Furthermore, they have shown that AuNPs modified multifunctional mesoporous silica (MCM-41) can be employed for detection probe development by immobilizing Ab2 and TB. Using such approach they detected AFP down to 0.05 pg/mL [57]. Metal alginate nanobeads (M-Alg), with different metals attached to specific detection antibodies can be employed for simultaneous estimation of biomarkers such as AFP, CEA and PSA [43]. Similarly Metal-Envision copolymer has also been utilized for detection probe preparation to achieve enhanced detection of Ca19-9, AFP and CEA [107].

Zhu et al. described the use of a hybridization chain reaction-based approach for testing four biomarkers simultaneously. For detection probe development, biotin-Ab2 was mixed with gold magnetic particles (Au/Sio_2_-Fe_3_O_4_). The conjugate was then treated in sequence with streptavidin bio-S1, bio-S2 and bio-S3 for bio-dsDNA/SA/bio-Ab2/Au/SiO_2_-Fe_3_O_4_ development via HCR reaction. Product was then modified with redox tag-streptavidin to obtain the detection probe. With such a probe they were able to detect AFP, CEA, CA125 and PSA down to 62, 48, 77 and 60 fg/mL, respectively [46].

A quadruple signal amplification strategy has been described by Zhou et al. for CEA detection. In amplification strategy streptavidin-labeled gold nanoparticles (AuNP-SA) were utilized for immobilizing detection antibody (Ab2) and initiator DNA strands (s0) using avidin-biotin coupling. For amplified electrochemical signal measurement, CEA sandwiched between Ab1 immobilized on sensor surface and modified Ab2 underwent hybridization with s1 and s2 DNA strands to form a concatamer followed by interaction with hemin, which resulted in formation of DNAzyme capable of binding with methylene blue. During DPV measurement, reduction of H_2_O_2_ by DNAzyme helped in enhancing methylene signal and sensor for CEA detection exhibited linearity in 1.0 fg/mL to 20 ng/mL range with detection limit of 0.5 fg/mL [60].

Zhang et al. described the development of a sensing strategy using signal tag of PtNP-ferrocenedicarboxylic acid based infinite coordination polymer (ICP) in combination with polyamidoamine dendrimers modified sensor electrode for PSA estimation in a sandwich type electrochemical ELISA. PtNP@ICP tag enhanced the catalytic reduction of H_2_O_2_ during the immunoassay to measure PSA. DPV measurements indicated that the sensor is able to detect PSA in the 0.001 to 60 ng/mL range with detection limit (LOD) of 0.3 pg/mL [61]. Shan and Ma described the development of a multiple probe by attaching desired Ab2 with specific redox tag and utilized for simultaneous detection of five biomarkers. Using PBG-Au, PPP-Au, PTBO-Au, PMCP-Au and Cd NCs-based probes they detected CEA, NSE, CA125, Cyfra21–1 and SCCA simultaneously at 0.4 V, 0.15 V, −0.14 V, −0.5 V and −0.75 V in SWV scans and achieved detection down to 0.2 ng/mL for CEA, 0.9 ng/mL for NSE, 0.9 U/mL for CA125, 0.4 ng/mL for Cyfra21–1 and 0.03 ng/mL for SCCA [80]. Zhu et al. described the use of primer-AuNP-PSA aptamer-based probe with RCA reaction-based approach for enhanced detection of PSA. During immunoassay captured primer-AuNP-PSA aptamer was utilized for RCA reaction and CuNP formation in presence of sodium ascorbate and copper sulphate. Formed CuNPs were then extracted in HNO_3_ and utilized for sensitive detection of PSA down to 0.02 fg/mL via DPSV measurements [83].

#### 3.2.2. Redox Marker with Carbon Material Based Enhancement

**Figure 8:**
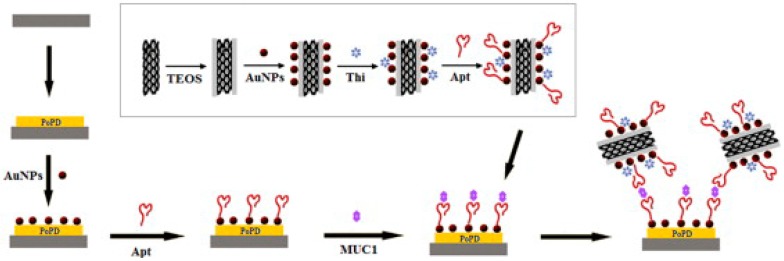
Illustration of the electrochemical sensing strategy for the detection of MUC 1. Inset: Preparation of Apt/Thi-AuNPs/SiO_2_@MWCNTs. Reproduced with permission from [58].

**Figure 9:**
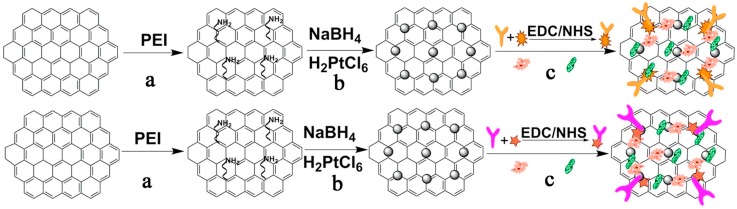
Preparation procedure of PGN-Ab1/2 probes: (a) modification with PEI to obtain active groups of amino; (b) reducing H_2_PtCl_6_ to form PtNPs; (c) labeling PGN with thionine-anti-CEA, ferrocene-anti-AFP, HRP and GOD. Reproduced with permission from [48].

Other than metallic nanomaterials, carbon materials in various forms such as graphene, CNTs, etc. have also gained much attention in enhancement strategies for electrochemical ELISA based assays. These materials provide support to load redox marker and detection antibody for signal enhancement. This section summarizes various such approaches described by researchers for enhancing the sensitivity of immunoassays. Using MWCNTs, Chen et al. described a AuNPs/SiO_2_@MWCNTs-based detection probe. For detection probe development, COOH-MWCNTs (c-MWCNTs) were first treated with PDDA to get positively charged MWCNTs, which were then treated with TEOS to make SiO_2_@MWCNTs. The obtained SiO_2_@MWCNTs were again treated with PDDA before incubating in AuNPs solution for 8 h to obtain a AuNPs/SiO_2_@MWCNTs nanocomposite. The composite was then incubated with thionine followed by an aptamer (Apt) solution, where Apt becomes covalently attached to AuNP via a thiol group. Using this probe, they were able to detect MUC1 down to 1 pM. Figure 8 illustrates the electrochemical sensing strategy for the detection of MUC 1, with the inset showing the preparation of Apt/Thi-AuNPs/SiO_2_@MWCNTs [58]. PtNPs modified graphene nanocomposites (PGN) were utilized by Jia et al. to develop detection probe for AFP and CEA detection. Using Fc-anti-AFP-PGN or Thi–anti-CEA-PGN they achieved detection down to 1.64 pg/mL and 1.33 pg/mL for CEA and AFP, respectively. Figure 9 illustrates the preparation procedure of PGN-Ab1/2 probes [48]. In another study, Li et al. described 3D graphene sheet (3DGS) prepared from GO reduction using NaI, based detection probe for CEA and AFP detection. On paper based assay with 3DGS@MB and 3DGS@Fc-COOH nanocomposites based probes resulted in CEA and AFP detection down to 0.5 and 0.8 pg/mL, respectively [76].

**Figure 10:**
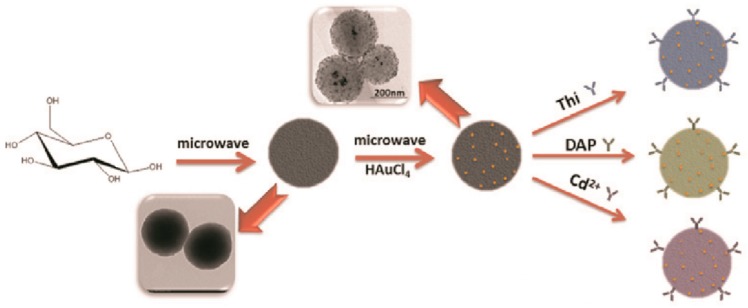
Schematic illustration of the electrochemical probe development. Reproduced with permission from [75].

Xu et al. described the development of carbon and gold (CGN) nanocomposite-based immunoprobes for simultaneous detection of multiple cancer marker. CGN was prepared via glucose carbonization in the presence of sodium citrate followed by microwave reaction-based AuNPs deposition from HAuCl_4_. Further, redox tags were attached using reactive oxygen groups on CGN via mixing and stirring for 5 h. Physically immobilized antibody-based CGN-Thi-anti-CEA, CGN-DAP-anti-PSA and CGN-Cd^2+^-anti-AFP probes showed detection limit of 4.8, 2.7 and 3.1 pg/mL for PSA, CEA and AFP, respectively. Figure 10 illustrates the electrochemical probe development [75].

#### 3.2.3. Non-Enzymatic Catalytic Activity and Enzyme-Mimicking Materials Based Signal Amplification Strategies

The following section describes various alternative techniques other than those based on redox enzymes or tags to enhance the response of immunosensors. To get better sensitivity and more stable sensor systems, recently there has been a great interest in the development of detection probes, which can show non-enzymatic catalytic activity, or which can mimic enzymatic behavior. Among this category, various materials like Pt NPs, Fe_3_O_4_, etc. exhibiting catalytic activity towards H_2_O_2_ have gained maximum attention. In such approach, Cui et al. described mesoporous platinum nanoparticles (M-Pt NPs) as a non-enzymatic label-based immunosensor strategy. Ab2 were immobilized physically and during assay M-Pt showed high catalytic activity toward added H_2_O_2_ and the sensor exhibited detection limits of 7.0 pg/mL, 0.001 U/mL and 0.002 U/mL, for CEA, CA153 and CA125, respectively [30]. In another study, Feng et al. described a ferrocene modified ferroferric oxide@silica–amino groups (Fe_3_O_4_@SiO_2_–NH_2_)-based strategy for signal enhancement and detection of CEA. 

**Figure 11:**
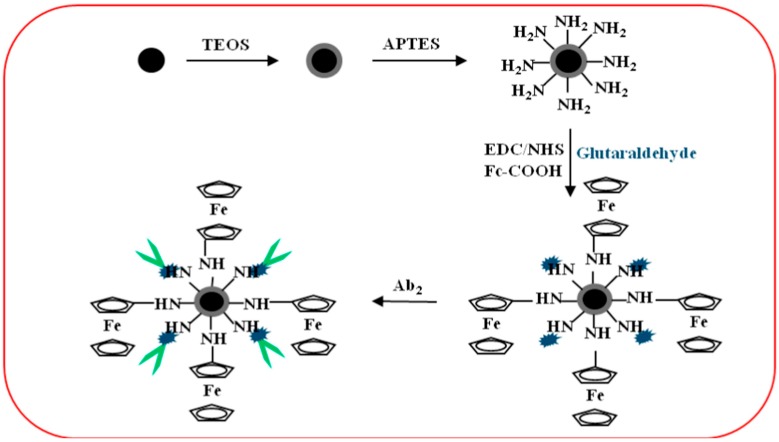
Preparation of Fe_3_O_4_@SiO_2_–Fc–Ab2/HRP bioconjugate. Reproduced with permission from [35].

For detection probe, Fe_3_O_4_ particles prepared by a solvothermal method were first treated with TEOS to obtain Fe_3_O_4_@SiO_2_ particles, which were then again treated with APTES to get Fe_3_O_4_@SiO_2_–NH_2_. For Fc-COOH and GA binding on prepared particles, Fc-COOH was first activated using EDC/NHS and then incubated with Fe_3_O_4_@SiO_2_–NH_2_ and GA overnight with stirring. Fe_3_O_4_@SiO_2_/Fc/GA precipitates thus obtained were utilized for Ab2 binding by incubation at 4 °C for 2 h. During immunoassay, Fe_3_O_4_ of captured probe provide catalytic activity towards H_2_O_2_, which in turn reduce Fc molecules and provide the detection signal. With such approach, they were able to detect CEA down to 0.0002 ng/mL. Figure 11 illustrates preparation of Fe_3_O_4_@SiO_2_–Fc–Ab2/HRP bioconjugate [35].

**Figure 12:**
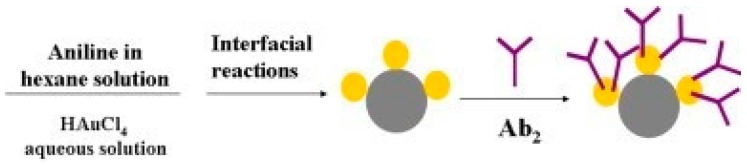
Schematic representation of the preparation of the PANi–Au AMNPs-Ab2. Reproduced with permission from [55].

**Figure 13:**
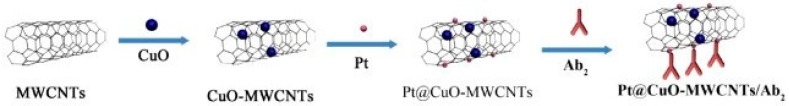
The preparation procedures of Pt@CuO-MWCNTs/Ab2. Reproduced with permission from [38].

A polyaniline–Au asymmetric multicomponent nanoparticles (PANI–Au AMNPs)-based strategy for immunosensor development was described by Fan et al. In their approach, captured PANI–Au AMNPs exhibited catalytic activity towards added H_2_O_2_ and the sensor showed CA72-4 detection down to 0.10 U/mL. Figure 12 shows the schematic representation of the preparation of the PANI–Au AMNPs-Ab2 [55]. Gao et al. described the use of Pd-Au/C-based probe for SCCA detection. During the immunoassay, Pd-Au helped in achieving higher signal from H_2_O_2_ during amperometric measurements and achieved detection down to 1.7 pg/mL [28]. The authors also proposed a Cu@Ag-CD-based enhancement strategy for immunosensor for CEA. In immunoassay Cu@Ag in H_2_O_2_ presence generated an enhanced signal and achieved detection down to 20 fg/mL [37]. A palladium nanoparticles/carbon-decorated magnetic microspheres-based strategy for development of immunosensor for AFP was described by Ji et al. In this probe Fe_3_O_4_@C@Pd generated an enhanced signal for H_2_O_2_ during amperometric measurement in the assay and achieved detection down to 0.16 pg/mL [33]. A Pt@CuO-MWCNTs based probe was described by Jiang et al. for AFP estimation. In presence of H_2_O_2_ Pt@CuO-MWCNTs catalyzed the reaction and generated enhanced signal for AFP detection down to 0.33 pg/mL. Figure 13 shows the preparation procedures of Pt@CuO-MWCNTs/Ab2 [38].

**Figure 14:**
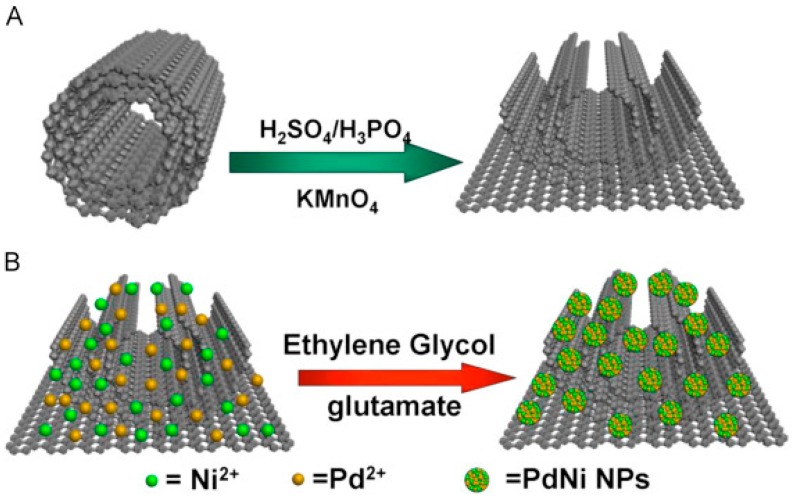
(**A**) The synthetic process of N-GNRs from N-MWCNTs; (**B**) The synthetic process of PdNi/N-GNRs. Reproduced with permission from [39].

**Figure 15:**
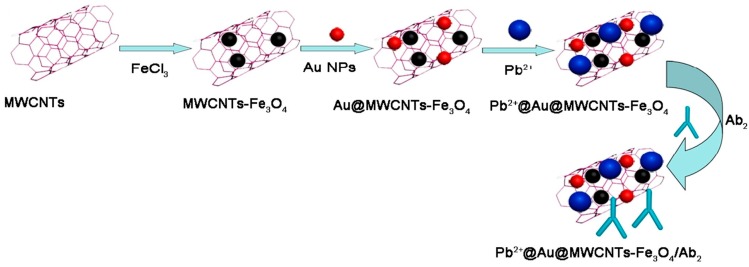
The preparation procedure of Pb^2+^@Au@MWCNTs-Fe_3_O_4_/Ab_2_. Reproduced with permission from [49].

Li et al. utilized nanoporous PtFe (NP-PtFe) alloys for probe development and enhanced catalytic conversion of H_2_O_2_ during amperometric measurement of CA15-3. With NP-PtFe, they achieved detection down to 3 × 10^−4^ U/mL when measured in the presence of 5 mM H_2_O_2_ at −0.4V [31]. Li et al. described PdNi/N-GNRs-based probe for H_2_O_2_ catalysis. N-GNRs were prepared via microwave-assisted method and modified with PdNi. During assay PdNi enhanced catalysis and helped obtaining a higher signal for AFP detection. With such a probe they achieved detection down to 0.03 pg/mL when measured in the presence of 5 mM H_2_O_2_ [39]. Figure 14 shows the synthetic process of N-GNRs from N-MWCNTs and the synthetic process of PdNi/N-GNRs. In another study Li et al. described the use of Pb^2+^@Au@MWCNTs-Fe_3_O_4_ for enhanced H_2_O_2_ catalytic conversion for AFP detection down to 3.33 fg/mL [49]. Figure 15 illustrates the preparation procedure of Pb^2+^@Au@MWCNTs-Fe_3_O_4_/Ab2.

Au/Ag/Au core/double shell nanoparticles (Au/Ag/Au NPs) as novel enzyme-mimetic labels for anti-SCCA have been described by Wang et al. The study showed that improved electrocatalytic activity of Au/Ag/Au NPs for H_2_O_2_ reduction resulted in enhanced sensitivity and detection of SCCA down to 0.18 pg/mL [41]. For AFP estimation, Wei et al. described the use of anti-AFP tagged GO-CeO_2_ and Pd nanoparticle-based probes. In such a Pd/APTES-M-CeO_2_-GS-based probe, Pd octahedral NPs showed enhanced catalytic activity for H_2_O_2_ reduction and the sensor achieved detection down to 0.033 pg/mL when measured amperometrically at −0.4 V in the presence of 5 mM H_2_O_2_ [51]. Dumbbell shaped Pt–Fe_3_O_4_ as labels were described by Wu et al. for SCC estimation. Results indicate that Pt–Fe_3_O_4_ improve H_2_O_2_ reduction and the immunosensor achieved linearity in the 0.05–18 ng/mL range with a detection limit of 15.3 pg/mL [65]. In other study, Wu et al. described the use of a dumbbell-shaped PtPd-Fe_3_O_4_ nanoparticles-based label in designing immunosensor for CA72-4 biomarker for gastric cancer. Results indicated that PtPd–Fe_3_O_4_ improve H_2_O_2_ reduction and immunosensor achieved linearity in the 0.001–10 U/mL range with detection limit of 0.0003 U/mL [67]. Li et al. described the mesoporous core-shell Pd@Pt nanoparticles loaded by amino group functionalized graphene (M-Pd@Pt/NH_2_-GS)-based detection. With such a probe, they achieved higher reduction of H_2_O_2_ to improve the sensitivity of the immunosensor. In immunoassay for PSA detection they achieved detection down to 3.3 fg/mL [78]. Miao et al. described the use of PVP-stabilized colloidal iridium nanoparticles, prepared via ethanol reduction-based detection probe. Physically immobilized anti-CEA was used for detection and Ir NP-based catalyzed reduction of H_2_O_2_ helped in achieving amperometric detection of CEA at −0.6 V down to 0.23 pg/mL [79]. 

Table 2 and Table 3 show the details of various strategies used for development of detection probes for enhanced detection and the various characteristics of the developed immunoassays using electrochemical ELISA, respectively.

## 4. Conclusions and Outlook

In the last few years, researchers have shown that electrochemical ELISA-based immunosensors can achieve similar or even better performance when compared to traditional optical ELISA immunoassays and are capable of replacing them in the near future. The innovations in nano- and bio-technologies and in surface and material chemistry have resulted in the development of novel sandwich assays with improved performance and stability. Further, due to the use of electrochemical techniques for testing, they have the advantage of providing faster response and on site testing in either undiluted or treated samples. This review has also described the various approaches which have been attempted by researchers to develop novel electrochemical immunosensors. It is clear that newer matrices and immobilization platforms allow higher capturing molecule loading and thus enhanced signals. Furthermore, the use of carrier materials for detection tags before electrochemical measurement helps in enhancing the sensitivity of the immunosensors. Although there are many success stories, there are a few limitations which need further detailed investigation before these electrochemical ELISA-based sensors can be accepted in clinical practice and able to replace optical ELISA. To improve the shelf life of the systems and to improve their stability, more detailed research is still required to understand the nature of biomolecule bound on flat matrices and on nanomaterials. Further, more studies are required for better surface blocking to prevent non-specific binding, while maintaining conductivity of sensor surface for higher electrochemical signal and better sensitivity. Moreover, newer and better packaging approaches are required to be developed to hold all the required chemicals and reagents required in a multistep ELISA processor, so that the assay can be automated and made less prone to human errors. It is envisioned that further advancements in nano- and bio-technology along with chemistry, material science, physics and electronics will pave the way to solve these issues and result in larger acceptance of these devises in clinical practice.

## Figures and Tables

**Table 1:** Various immunosensors and their preparation conditions.

Immunosensor	Components	Preparation Conditions	Binding Technique	Target	Ref.
Ab1/GS/SPCE	GS	Acid treatment of graphite flakes	EDC/NHS (covalent)	CEA, CA125, CA153	[30]
Ab1/N-GS/GCE	N-GS	GO prepared from acid treatment of graphite and then reduced with DMF at 153 °C to get N-GS	glutaraldehyde (covalent)	SCCA	[28]
Ab1-ADA/CD-GN/GCE	Ab1-ADA	EDC/NHS chemistry	physical	CEA	[37]
	CD-GN	GO prepared from acid treatment of graphite and then reduced with hydrazine in presence of ammonia and β-CD at 60 °C to get CD-GN			
Ab1/MWCNTs/GCE	MWCNT-COOH	Acid treatment of MWCNTs	EDC/NHS (covalent)	AFP	[33]
Ab1/CD-GS/GCE	CD-GS	GO prepared from acid treatment of graphite and then reduced with hydrazine in presence of ammonia and β-CD at 180 °C to get CD-GS	physical	AFP	[38]
Ab1-GS/GCE	GS	GO prepared from acid treatment of graphite flakes and then reduced with hydrazine at 100 °C to get GS	EDC/NHS (covalent)	CA 15-3	[31]
Ab1-ADA/CD-GS/GCE	Ab1-ADA	EDC/NHS chemistry	EDC/NHS (covalent)	AFP	[39]
	CD-GS	GO prepared from acid treatment of graphite and then reduced with hydrazine hydrate in presence of ammonia and β-CD at 60 °C to get CD-GS			
Ab1-PA/AuNP/CSSH-SWCNTs/Au	AuNPs	Sodium citrate based reduction at 100 °C	PA-antibody interaction	AFP	[40]
	CSSH	EDC/NHS chemistry			
anti-HER2 Nb/SPE	COOH-SPE	Acid treatment at 1.6 V	EDC/NHS (covalent)	HER2	[63]
Ab1/GS-Thi/GCE	GS-Thi	Thi adsorption on GS	EDC/NHS (covalent)	AFP	[64]
Ab1/IL-rGO/GCE	IL-rGO	treating GO with IL-NH_2_ in KOH at 80 °C	glutaraldehyde (covalent)	CEA, AFP	[29]
Ab1/N-GS-CH/GCE	N-GS-CH	GO prepared from acid treatment of graphite and then undergo thermal annealing in ammonia to get N-GS which was then mixed with CH to get N-GS-CH	glutaraldehyde (covalent)	SCC	[65]
Ab1/rGO-TEPA/GCE	rGO-TEPA	rGO-TEPA	EDC/NHS (covalent)	CEA, SCCA	[66]
Ab1/rGO-TEPA/GCE	rGO-TEPA	rGO-TEPA	EDC/NHS (covalent)	CA72-4	[67]
Ab1-GS/GCE	GS-COOH	GO prepared from acid treatment of graphite and then undergo thermal exfoliation in quartz tube at 1000 °C to get GS which was then treated chloroacetic acid in basic media to generate GS-COOH	EDC/NHS (covalent)	CA15-3	[32]
Ab1/MWCNTs/DAH/GCE	MWCNT-COOH	Nitric acid treatment of MWCNTs	EDC/NHS (covalent)	PSA	[68]
	DAH monolayer	CV scans in 0.2 and 1.6 V at 20 mV/s			
Ab1/CH/rGO/SPC/whatman paper	rGO	GO prepared from acid treatment of graphite and reduced electrochemically at −1.0 V CH coating	glutaraldehyde (covalent)	AFP, CEA, CA125, CA153	[69]
Ab1/nafion-AuNP-DN-GR/GCE	GR	GO prepared from acid treatment of graphite and reduced using NaBH_4_ at 85 °C	physical	CEA	[34]
	AuNPs	Sodium citrate based reduction at 97 °C			
Ab1/thionine/AuNP-PMMA dendrimer/CH-MWCNTs-IL/GCE	AuNP-PMMA dendrimer	AuNPs prepared via citrate method were mixed and incubated with thiol terminated PAMAM prepared via treating amine-terminated PAMAM dendrimer (G4) with methyl mercaptoacetate at 50 °C	phthaloyl chloride (covalent)	PSA	[42]
Ab1-thionine/CH/rGO/GCE	rGO	GO prepared from acid treatment of graphite and reduced electrochemically at −1.0 V CH coating	glutaraldehyde (covalent)	PSA	[70]
Ab1/Au@SH-GS/GCE	AuNPs	Sodium citrate based reduction in boiling condition	physical	SCCA	[41]
	SH-GS	GO prepared from acid treatment of graphite was treated with MPTES at 70 °C followed by treating with hydrazine hydrate at 95 °C to get SH-GS			
Ab1/AuNPs-IL-rGO/GCE	IL-rGO	GO was mixed with IL-NH_2_ in KOH and reflux at 80 °C to get IL-rGO	physical	AFP, CEA, PSA	[43]
	AuNPs-IL-rGO	IL-rGO mixed with HAuCl_4_ was reduced using ascorbic acid to get AuNPs-IL-rGO nanocomposite			
Ab1/Au@APTES-GS/GCE	APTES-GS	GO treated with APTES at 70 °C followed by treating with hydrazine hydrate at 95 °C to get APTES-GS	physical	CEA	[45]
	AuNPs	HAuCl_4_ reduction using NaBH_4_ in ice bath			
Ab1/CH/rGO/GCE	rGO	GO prepared from acid treatment of graphite and reduced electrochemically at −1.0 V after CH coating	glutaraldehyde (covalent)	CEA	[71]
Ab1/AuNPs-IL-rGO/GCE	IL-rGO	GO was mixed with IL-NH_2_ in KOH and reflux at 80 °C to get IL-rGO	physical	CEA, AFP	[44]
	AuNPs	HAuCl_4_ reduction using NaBH_4_ and/or sodium citrate			
Ab1/Au-GR/GCE	Au-GR	Mix HAuCl_4_ with GO and perform 5 CV scan in −1.5 V to 0 V at 50 mV/s	physical	AFP, CEA, CA125, PSA	[46]
Ab1/Au-PGO/GCE	Au-PGO	Treat GO, HAuCl_4_ and PEG mixture at 180 °C	physical	CA19-9	[72]
Ab1/Au-Gra/GCE	Au-Gra	Treat GO-AA mixture with HAuCl_4_ at room temperature	physical	AFP	[73]
Ab1/AuNPs/CH-Thi-CNTs/GCE	AuNP	Electrochemical deposition at −200 mV	physical	CEA	[47]
	Thi-CNT	Modify COOH-CNTs with thionine using EDC/NHS chemistry			
Ab1/GO-AuNP/GCE	GO AuNP		physical	CEA	[35]
Ab1/HAG/PANI/rGO/GCE	HAG	Electrochemically deposited at −200 mV	physical	CEA, AFP	[36]
	PANI	Electro-polymerization at 0.75 V			
Ab1/CH-AuNP/GCE	CH-AuNPs	NaBH_4_ based reduction of CH-HAuCl_4_ solution	EDC/NHS (covalent)	CEA, AFP	[52]
Ab1/NPG/GCE	NPG	Acid based removal of silver from silver gold alloy	physical	CA72-4	[55]
Ab1/AuNPs/GCE	AuNPs	Electrodeposited at −0.2V	physical	CEA, AFP	[48]
Ab1/AuNPs/GCE	AuNPs	Electrodeposited at −0.2V	physical	AFP	[49]
Ab1/AuNPs/GCE	AuNPs	Sodium citrate based reduction in boiling condition	physical	AFP	[50]
	AuNPs/GCE	Electrochemical deposition at 1.5 V			
Ab1/CH-AuNP/GCE	CH-AuNPs	NaBH_4_ based reduction of CH-HAuCl_4_ solution	Physical	CEA, AFP	[53]
Ab1/MoS_2_-Au/GCE	MoS_2_-Au	Citrate based reduction of HAuCl_4_-MoS_2_ nano-sheets solution	Physical	CEA	[56]
Ab1-TB/Au@MCM-41/GCE	NH_2_-MCM-41	Treating MCM-41 with APTES at 70 °C	physical	AFP	[57]
	AuNPs	HAuCl_4_ reduction using NaBH_4_ in ice bath			
Ab1/CH-AuNPs/GCE	CH-AuNPs	Refluxing CH-HAuCl_4_ solution for 1 h	glutaraldehyde (covalent)	CEA, AFP	[54]
Ab1-biotin/streptavidin/Au–Fe_3_O_4_@SiO_2_/Au/magnet	Fe_3_O_4_	Treating FeCl_2_, FeCl_3_, and PEG 4000 mixture with NaOH at 80 °C	streptavidin-biotin interaction	CA 19-9	[74]
	Fe_3_O_4_@SiO_2_	Treating PDDA-Fe_3_O_4_ solution pH 11 (using ammonia) with TEOS at room temperature			
	Au–Fe_3_O_4_@SiO_2_	Treating PDDA-Fe_3_O_4_@SiO_2_ solution with AuNPs solution			
Ab1/PSS/IL-rGO/GCE	IL-rGO	GO was mixed with IL-NH_2_ in KOH and treated at 80 °C	electrostatic	CEA, PSA, AFP	[75]
aptamer/AuNP/oPD/Au	oPD/Au	Electropolymerized via CV scans in −0.5 and 0.8 V range at 50 mV/s	physical	MUC 1	[58]
Ab1/AuNPs/GCE	AuNPs	Electrodeposited at −0.2 V	physical	AFP	[51]
Ab1/PANI/Au/paper	Au	Seed layer using AuNPs prepared via NaBH_4_, citrate method; Au layer using growth solution of HAuCl_4_ cetyltrimethyl ammonium chloride	glutaraldehyde (covalent)	CEA, AFP	[76]
	PANI	20 CV scans in −0.1 to 0.8 V range at 50 mV/s			
Ab1/β-CD/GCE	oxidize GCE	5 CV scans in H_2_SO_4_ solution in 0 to 2 V	physical	CEA	[60]
Ab1/PAMAM/GCE	PAMAM/GCE	Using infrared light treatment	EDC/NHS (covalent)	PSA	[61]
Ab1/cysteine /Au	cysteine /Au	Self-assembled monolayer	EDC/NHS (covalent)	CEA	[62]
PSA aptamer/GDPTS/PDMS	GDPTS/PDMS	Self-assembled monolayer	epoxide chemistry	PSA	[77]
Ab1/Au@MWCNTs-SO_3_H/GCE	MWCNTs-SO_3_H	Refluxing MWCNTs in H_2_SO_4_-HNO_3_ at 120 °C, 30 min	physical	PSA	[78]
	AuNPs	Sodium citrate based reduction at 100 °C reflux			
Ab1/PDA-rGO/GCE	PDA-rGO	Mixing dopamine with GO and stirring for 24 h at 25 °C	physical	CEA	[79]
Ab1/AuNPs/GCE	AuNPs	Electrodeposited at −0.2 V	physical	CEA, NSE, CA125, Cyfra21–1, SCCA	[80]
Ab1/MPA/Au	MPA/Au	Self-assembled monolayer	EDC/NHS (covalent)	PSA, PSMA, IL-6, PF-4	[81]
Ab1/MUDA-mercapto ethanol/Au	MUDA-mercapto ethanol	Self-assembled monolayer	EDC/NHS (covalent)	PSA	[82]
Ab1/PS			physical	PSA	[83]
Ab1/3D-G-CH/GCE	3D-G	GO was first prepared from natural graphite powder by Hummer’s method followed by autoclaving at 180 °C to get 3D-G. Dried 3D-G was then mixed in 1% CS	glutaraldehyde (covalent)	CYFRA21-1	[84]
Ab1/polystyrene; AgNP/SPCE	AgNPs	Sodium citrate-based reduction of AgNO_3_ in boiling condition	physical	AFP	[85]
antiHER2/APTMS-Fe_3_O_4_/GCE	Fe_3_O_4_	Chemical co-precipitation from FeCl_3_·6H_2_O and FeCl_2_·4H_2_O mix using ammonia solution	glutaraldehyde (covalent)	HER2	[86]
Anti-CEA/LPA/Au	NHS-LPA/Au	Self-assembly	covalent	CEA	[87]
Ab1-AuNPs/CHI/SPE	AuNPs	Electrochemical reduction in 0.5 M H_2_SO_4_ via CV scans between −1.5 and 0.5 V at a rate of 30 mV/s	physical	PSA	[88]
BSA/anti-CEA/AuNPs/GCE	AuNPs	Electrodeposit deposition by cyclic sweeping in the potential range of −0.5 to 0 V (vs. SCE) at 50 mV/s for 50 segments	physical	CEA	[89]
Ab1/Au@Th/GO/GCE	Au@Th/GO	GO synthesized using modified Hummers’ method was mixed with Thi and HAuCl_4_ solution and stir	physical	PSA	[90]
Ab1/Au@MWCNTs-SO_3_H/GCE	AuNPs	Citrate reduction of HAuCl_4_ solution;	physical	PSA	[78]
	Au@MWCNT-SO_3_H	Physical adsorption of AuNPs on MWCNTs-SO_3_H			
Ab1/Au@MPTES-GS/GCE	AuNPs	Citrate reduction of HAuCl_4_ solution	physical	AFP	[91]
	MPTES-GS	GO synthesized using modified Hummers’ method was treated with MPTES in ethanol at 70 °C for 2 h followed by treatment with hydrazine solution at 95 °C for 1.5 h			
Ab1/AuNPs/GCE	AuNPs	Electrochemical reduction at −0.2 V, 30 s	physical	CEA	[92]
Ab1/β-CD/MWCNT/GCE	β-CD/MWCNTs	Grind rMWCNTs and β-CD in ethanol	physical	CEA	[93]
Ab1/streptavidin-NG-CH/GCE	NG-S	GO synthesized using modified Hummers’ method was refluxed with hydrazine at 100 °C, 24 h. Obtained rGO was then mixed with pyrrole and treated with ammonium peroxydisulphate. Obtained PPY-rGO was heat treated till 600 °C, 2 h	Biotin-streptavidin		[94]
Ab1/AuNPs/GCE	AuNPs	Electrochemical reduction at −0.2V, 30 s	physical	CEA	[95]
Fe_3_O_4_@AuNPs-Ab1	Fe_3_O_4_	From ferrous complex via hydrothermal method using H_2_O_2_ as oxidizer	physical	AFP	[96]
	Fe_3_O_4_@AuNPs	Mixture of Fe_3_O_4_ NPs with PEG 20000 and HAuCl_4_ was treated with hydroxylamine hydrochloride			
Ab1/Au/ZnO/RGO/GCE	Au/ZnO/RGO	GO synthesized using modified Hummers’ method was mixed with C_12_N_3_. Solution was adjusted to pH 12 and mixed with Zn(NO_3_)_2_ and HAuCl_4_ followed by treatment with hydrazine at 105 °C, 5 h	physical	AFP	[97]
Ab1/CH/CNT/SPE	CH/CNT/SPE	Acid treated CNTs were mixed with nafion 117 and drop casted on SPE followed by deposition of CH solution	glutaraldehyde (covalent)	PSA	[98]
Ab1/AuNP/GCE	AuNPs	Electrochemical reduction at −0.2 V, 30 s	physical	SCCA	[99]
Ab1/AuNP/GCE	AuNPs	Electrochemical reduction at −0.2 V, 30 s	physical	AFP	[100]
Ab1-BSA/AuNP/PANI/GCE	PANI/GCE	Phytic acid doped polyaniline via electrochemical co-deposition at 0.8 V, 400 s	physical	PSA	[101]
	AuNPs	Electrodeposit deposition by cyclic sweeping in the potential range of −1 to 0.2 V at 50 mV/s, 10 cycles			
Ab1/AuPd NCNs/GCE	AuPd NCNs	Add HAuCl_4_, H_2_PdCl_4_ and PVP sequentially into NaOH solution containing T7AA	physical	CA 15-3	[102]
Ab1/Au@PDA/GCE	Au@PDA	Citrate reduced AuNPs were treated with dopamine in tris buffer	physical	CEA	[103]
Ab1/Au@N-GQD/GCE	N-GQD	Dicyandiamide and CA solution was autoclaved at 180°C, 12 h	physical	PSA	[104]
	Au@N-GQD	HAuCl_4_ was added to N-GQD, pH adjusted to 10 using NaOH followed by autoclaving at 160 °C, 6 h			

Notes: β-CD: β-cyclodextrin; 3D-G: 3-dimensional graphene; AA: ascorbic acid; Ab1: capture antibody; ADA-COOH: adamantine-1-carboxylic acid; AFP: α-fetoprotein; APTES: 3-aminopropyltriethoxysilane; APTMS: 3-aminopropyltrimethoxysilane; CA: citric acid; CA 19-9: carbohydrate antigen 19-9; CA125: carbohydrate antigen 125; CA15-3: carbohydrate antigen 15-3; CEA: carcinoembryonic antigen; CH: chitosan; CSSH: L-cysteine modified chitosan; CV: cyclic voltammetry; Cyfra21–1: cytokeratin 19 fragment antigen 21–1; DAH: 1, 7-diaminoheptane; DMF: dimethylformamide; DN: 1,5-diaminonaphthalene; EDC: 1-Ethyl-3-(3-dimethylaminopropyl)-carbodiimide; GDPTS: (3-glycidyloxypropyl) trimethoxysilane; GN: graphene nanosheet; GS: graphene sheet; IL-6: interleukin-6; IL-NH_2_: 1-aminopropyl-3-methylimidazolium chloride; LPA: lipoic acid N-hydroxysuccinimide ester; MCM-41: multifunctional mesoporous silica; MPA: mercaptopropionic acid; MUDA: mercaptoundecanoic acid; Nb: nanobody (antigen-binding fragments with single domain); N-GS: nitrogen doped graphene sheet; NHS: N-Hydroxysuccinimide; NPG: nanoporous gold; NSE: neuron specific enolase; oPD: poly(o-phenylenediamine); PA: protein A; PAMAM: polyamidoamine dendrimers; PDA: polydopamine; PDDA: poly(diallyldimethylammonium chloride); PF-4: platelet factor-4; PGO: porous graphene oxide; PS: polystyrene; PSA: prostate specific antigen; PSMA: prostate specific membrane antigen; PSS: poly(sodium-p-styrenesulfonate); SAM: self-assembled monolayer; SCCA: squamous cell carcinoma antigen; SH-GS: mercapto-functionalized graphene sheets; SPC: screen printed carbon; SPCE: screen printed carbon electrode; TB: toluidine blue; TEPA: tetraethylene pentamine; Thi: thionine.

**Table 2:** Strategies used for the development of detection probes for enhanced detection.

Detection Probe	Components	Preparation Conditions	Ref.
HRP, Anti-CEA/AuNPs-PAN@CNTs	PAN@CNTs	(NH_4_)_2_S_2_O_8_ based polymerization of CNTs and aniline monomers solution in HCl at ice bath	[47]
	AuNPs	Citrate reduction	
	AuNPs-PAN@CNTs	Electrostatic assembly of AuNPs	
HRP-PSA aptamer/AuNP-PAMAM	AuNP	Citrate reduction	[70]
	thiol-PAMAM	Treating amine-terminated PAMAM dendrimer (G4) with methyl mercaptoacetate at 50 °C for 18 h	
	AuNP-PAMAM	Incubation for 5 h at RT	
Thi-Anti-AFP/HRP NPs-hollow AuNPs	hollow AuNPs	HAuCl_4_ reduction in N_2_ environment using NaBH_4_, sodium citrate and CoCl_2_·6H_2_O mixed solution	[40]
	HRP NPs-hollow AuNPs	Self-assembly of L-cysteine modified HRP-NPs prepared via desolvation followed by glutaraldehyde chemistry;	
	Thi-Anti-AFP	EDC/NHS chemistry	
HRP, anti-AFP/Fe_3_O_4_ NPs-MSNs	Fe_3_O_4_ NPs-MSNs	Treating APTES modified MSNs with bromine-functionalized Fe_3_O_4_ NPs in EtOH	[64]
AgNPs-GOx-anti CEA	AgNPs	Ag nanospheres prepared via ethylene glycol (EG) and poly(vinyl pyrrolidone) (PVP) based reduction	[56]
HRP-anti CA 19-9/Au@SBA-15	Au@SBA-15	incubating PDDA coated SBA-15 particles with AuNPs solution	[74]
Anti-CEA/Ag/Au–DN–GR	Ag/Au–DN–GR	(i) HAuCl_4_, AgNO_3_, trisodium citrate dihydrate and SDS mixture reduction using NaBH_4_, (ii) Mix and incubate Ag/Au with DN-graphene	[34]
PAMAM-Gr/anti-AFP-HRP	PAMAM-Gr	EDC/NHS chemistry	[50]
HRP, GOD, anti-AFP/SWCNHs	Carboxylated SWCNHs	Acid treatment of SWCNHs	[73]
AuNPs-MCF	carboxy-MCF	Refluxing MCF in acid	[71]
	AuNPs-MCF	NaBH_4_ based reduction of HAuCl_4_-MCF mixture	
CHIT-PB-AuNP CHIT-FC-AuNP	CHIT-PB	Treating K_3_Fe(CN)_6_ and FeCl_3_ solution (pH 1.5) in CHIT	[52]
	CHIT-FC	EDC/NHS chemistry;	
	AuNP binding	Physical adsorption by mixing	
anti-AFP_2,2_-AuNPs-Thi@rGO	AuNPs-Thi@rGO	(i) Incubating rGO and Thi for 12 h, (ii) Incubating Thi@rGO with AuNPs for 24 h, (iii) Mixing FeCl_3_ and K_3_Fe(CN)_6_ solution (pH 1.5) to rGO dispersion	[36]
anti-CEA_2,1_-AuNPs-PB@rGO	AuNPs-PB@rGO	(i) Mixing PB@rGO with PDDA, 30 min, (ii) Incubation with AuNPs	
Anti-CEA /PB–CS-Au and Anti-CEA/Cd–CS-Au	PB–CS-Au and Cd–CS-Au	(i) PBNPs and CdNPs were prepared using FeCl_3_ and CdCl_2_ were first mixed with CS solution in 1% acetic acid, (ii) Incubating PBNPs and CdNPs with AuNPs	[44]
PLL-Au-Cd-Apo-Ab2 and PLL-Au-Pb-Apo-Ab2	PLL-Au	Incubating PLL with citrate reduced AuNPs	[59]
	Cd-Apo and Pb-Apo	Dropwise adding metal ions (Cd^2+^, Pb^2+^) to Apo solution pH 2 and adjusting pH to 8.5 before stirring for 3 h	
anti-CEA/PtPNPs-Cd^2+^ and anti-AFP /PtPNPs-Cu^2+^	PtPNPs-Cd^2+^ and PtPNPs-Cu^2+^	(i) PtPNPs synthesised from chloroplatinic acid treatment with ascorbic acid in KOH, (ii) Mercapto-ethylamine modification of PtPNPs for capture of Cd^2+^ or Cu^2+^ ions	[29]
CdNCs–Au–anti-CEA and CuNCs– Au–anti-AFP	CdNCs–Au and CuNCs–Au	(i) Treating CdCl_2_ or CoCl_2_ in presence of CS with K_3_Co(CN)_6_ dispersed PDDA, (ii) Nanocubes incubation with AuNPs prepared via citrate and NaBH_4_ reduction	[53]
TB/Au@KIT-6/CMC/ILs-anti-CEA	Au@KIT-6	Treating APTES silanized KIT-6 with AuNPs prepared via NaBH_4_ reduction;	[45]
	TB/Au@KIT-6/CMC/ILs-Ab2	(i) Physical binding of anti-CEA on Au@KIT-6, (ii) TB binding using EDC NHS, (iii) Mixing and incubating with 1-butyl-pyridine tetrafluoroborate (ILs) dissolved in CMC	
Ab2/M-Alg; (M: Cd, Pb and Cu)	M-Alg	(i) Emulsion A: agitate the mixture of triton x-100, 1-hexyl alcohol, n-octane and sodium for more than 30 min RT, (ii) Emulsion B: agitate mixture of triton x-100, 1-hexyl alcohol, n-octane and Metal salt for more than 30 min RT, (iii) Add emulsion A dropwise to emulsion B and stir for 4 h RT, (iv) Break M-Alg using acetone and ethanol to get M-Alg nanobeads	[43]
M/Ab2-Envision copolymer; (M: AuNPs, CdS and PbS)	Ab2-Envision copolymer	Mix and incubate Ab2 with Envision (highly branched polymer) at 4 °C, 24 h	[107]
	M/Ab2-Envision copolymer	AuNP tagging via physical adsorption, CdS and PbS were bound to HRP modified envision-Ab2 via EDC/NHS chemistry	
Au@MCM-41/TB/Ab2	Au@MCM-41	APTES modified MCM-41 was mixed with AuNPs prepared via NaBH_4_ based reduction of HAuCl_4_	[57]
Au@CMK-3-anti-CEA-neutral red and Au@CMK-3-anti-SCCA-thionine	Au@CMK-3	AuNPs were prepared from sodium citrate based reduction of HAuCl_4_ were mixed and stir with mesoporous carbon CMK-3 for 4 h	[66]
AuNPs-Ab2-Cu^2+^ or Pb^2+^	AuNPs	Via sodium citrate based reduction of HAuCl_4_	[54]
	Cu^2+^ and Pb^2+^ tagging	Cu(NO_3_)_2_ or Pb(NO_3_)_2_ incubation with AuNPs-Ab2	
Redox tag bio-dsDNA/SA/bio-Ab2/Au/SiO_2_-Fe_3_O_4_	Au/SiO_2_–Fe_3_O_4_	(i) Nano-sized Fe_3_O_4_ via treating FeCl_2_-FeCl_3_ mixture with NaOH, (ii) Fe_3_O_4_–SiO_2_ via alkaline hydrolysis of TEOS, (iii) Au/SiO_2_–Fe_3_O_4_ via mixing and incubating PDDA treated SiO_2_–Fe_3_O_4_ with for 20 min	[46]
	bio-dsDNA/SA/bio-Ab2/Au/SiO_2_-Fe_3_O_4_	(i) Incubation of biotin-Ab2 Au/SiO_2_-Fe_3_O_4_ 12 h, 4 °C, (ii) Treatment with streptavidin (SA), initiator bio-S1, bio-S2 and bio-S3 in sequence to form bio-dsDNA/SA/bio-Ab2/Au/SiO_2_-Fe_3_O_4_ via HCR reaction	
Anti-CA15-3–f-TiO_2_–Cd^2+^	nanoporous TiO_2_	(i) Mixing and stirring tetrabutoxytitanium (TBOT) and ethylene glycol for 8 h, RT, (ii) Pouring mixture in acetone-water followed by vigorous stirring 1 h, (iii) Ethanol wash and drying at 50 °C, (iv) Mix with water and reflux for 1 h	[32]
	f-TiO_2_–Cd^2+^	(i) APTMS treatment to get NH_2_ functionalized nanoporous TiO_2_ (f-TiO_2_), (ii) Mixing f-TiO_2_ with Cd(NO_3_)_2_ and stirring for 24 h at 50 °C	
Anti-PSA/Fc-AuNPs	Fc-AuNPs	Self-assembly of 6-ferrocenyl hexanethiol onto AuNPs	[68]
Apt/Thi-AuNPs/SiO_2_@MWCNTs	Apt/Thi-AuNPs/SiO_2_@MWCNTs	(i) Treat COOH-MWCNTs (c-MWCNTs) with PDDA, (ii) TEOS modification to get SiO_2_@MWCNTs, (iii) Treatment with PDDA, (iv) Incubation in AuNPs solution to obtain AuNPs/SiO_2_@MWCNTs, (v) Mixing and incubating with thionine 1 h, RT, (vi) Incubation with SH-Apt solution	[58]
Ab2-PGN	rGO	Mix and refluxing GO with PEI	[48]
	PGN	Mix H_2_PtCl_6_ with rGO and treat with NaBH_4_	
Anti-CEA/APTES/3DGS@MB and anti-AFP/APTES/3DGS@Fc-COOH	3DGS	NaI based reduction of GO prepared from graphite	[76]
	APTES/3DGS@MB, APTES/3DGS@Fc-COOH	(i) Redox tag (MB for CEA and Fc-COOH for AFP) modification by mixing and stirring; (ii) Treatment with APTES to get amino functionalized composites	
CGN-Thi-anti-CEA, CGN-DAP-anti-PSA and CGN-Cd^2+^-anti-AFP	CGN	(i) Glucose carbonization in presence of sodium citrate, (ii) AuNPs deposition on carbon particles from HAuCl_4_ using microwave reaction	[75]
	Thi or DAP or Cd^2+^/CGN	Mixing Thi or DAP or Cd(NO_3_)_2_ with CGN and stirring for 5 h	
M-Pt-Ab2	M-Pt	Ascorbic acid based reduction of K_2_PtCl_4_	[30]
Anti-CA72-4/PANi–Au AMNPs	PANi–Au AMNPs	Mix and incubate aniline in hexane with HAuCl_4_ aqueous solution at 45 °C overnight	[55]
Fe_3_O_4_@SiO_2_/Fc/GA/anti-CEA	Fe_3_O_4_@SiO_2_/Fc/GA/	(i) Fe_3_O_4_ via solvothermal method; (ii) Treatment with TEOS to obtain Fe_3_O_4_@SiO_2_; (iii) Treatment with APTES to get Fe_3_O_4_@SiO_2_–NH_2_; (iv) Treatment with EDC/NHS activated Fc-COOH followed by treatment with GA	[35]
Anti-SCCA/Pd–Au/C	Pd–Au/C	Mixing activated carbon, PdCl_2_, HAuCl_4_ and H_2_O-tetrahydrofuran via ultra-sonicating and stirring followed by treatment with NaBH_4_ and Na_2_CO_3_	[28]
Cu@Ag-CD/anti-CEA	Cu@Ag-CD	CD-ascorbic acid (pH 11) solution based sequential reduction of CuSO_4_·5H_2_O and AgNO_3_ solution in ammonia, followed by mixing and stirring with HS-β-CD overnight; Obtained Cu@Ag-CD was used for EDC/NHS based binding of Ab2 modified ADA-COOH	[37]
Fe_3_O_4_@C@Pd/anti-AFP	Fe_3_O_4_@C@Pd	(i) Fe_3_O_4_@C magnetic nanoparticles via hydrothermal process; (ii) Treatment with PDDA followed by mixing and incubation with PDNPs prepared via citrate and NaBH4 based reduction of Na_2_PdCl_4_	[33]
Anti-CA15-3/NP-PtFe	NP-PtFe	By removing Al using NaOH from ternary PtFeAl alloy with 80%Al	[31]
Anti-AFP/PdNi/N-GNRs	PdNi/N-GNRs	(i) N-GNRs powders via microwave-assisted treatment of N-MWCNTs, (ii) Mix N-GNRs with aqueous solution of Na_2_PdCl_4_, NiCl_2_·6H_2_O, and glutamate in ethylene glycol (EG), (pH 11) and stirring it for 2h followed by heating at 160 °C for 6 h in autoclave to get PdNi/N-GNRs	[39]
Pb^2+^@Au@MWCNTs-Fe_3_O_4_/anti-AFP	Pb^2+^@Au@MWCNTs-Fe_3_O_4_	(i) MWCNTs-Fe_3_O_4_ via autoclaving the mixture of acid treated MWCNTs, FeCl_3_.6H_2_O and sodium acetate, (ii) Amino-functionalization via APTES modification, (iii) Mixing and incubation with AuNPs prepared via citrate reduction; (iv) Treatment with lead nitrate solution 24 h to get Pb^2+^@Au@MWCNTs-Fe_3_O_4_	[49]
Au/Ag/Au@anti-SCCA	Au/Ag/Au	(i) Mix AuNPs, ascorbic acid, and AgNO_3_ in CTAB solution, (ii) Add NaOH dropwise with vigorous stirring to get yellow-golden colored, silver coated Au particles, (iii) Mix with HAuCl_4_ and ascorbic acid and stirred vigorously to obtain dark-blue Au/Ag/Au NPs solution	[41]
Anti-AFP/Pd/APTES-M-CeO_2_-GS	Pd/APTES-M-CeO_2_-GS	(i) The Pd octahedral NPs via sonicating followed by heating the mixture of 1-ethenyl-2-pyrrolidinone homopolymer (PVP), citric acid, and Na_2_PdCl_4_ dissolved in ethanol and water at 80 °C with stirring and refluxing for 3 h, (ii) M-CeO_2_-GS prepared by dissolving Ce(NO_3_)_3_·6H_2_O into water followed by adding C_2_H_5_COOH, ethylene glycol and GO and then treating at 180 °C for 200 min followed by cooling, centrifuging the ppt and drying at 50 °C for 12 h, (iii) APTES modification of M-CeO_2_-GS by refluxing, (iv) Pd binding by sonication and stirring to get Pd/APTES-M-CeO_2_-GS	[51]
Anti-SCC-Pt–Fe_3_O_4_	Pt–Fe_3_O_4_	(i) Mix platinum acetylacetonate, oleic acid, oleylamine and octadecane under argon atmosphere followed by heating to 120 °C, (ii) add Fe(CO)_5_ heat at 280 °C, 20 min, (iii) Precipitation using ethanol addition	[65]
Anti-AFP/Pt@CuO-MWCNTs	Pt@CuO-MWCNTs	(i) Acid treated MWCNTs mixed with Cu(CH_3_COO)_2_·H_2_O were grounded and calcinated at 350 °C in argon, followed by addition of NH4OH solution, (ii) MWCNTs addition followed by ageing and calcination to get CuO/MWCNTs composite, (iii) Pt loading by adding CuO/MWCNTs nanocomposites to K_2_PtCl_4_ solution followed by Pt salt reduction	[38]
M-Pd@Pt/NH_2_-GS/anti-PSA	NH_2_-GS	(i) Mix GO and ethylene glycol under ultrasonication followed by ammonia water addition, (ii) Autoclave for solvothermal reaction at 180 °C, 10 h	[78]
	M-Pd@Pt	(i) Mix Pluronic F127 with aqueous solution of K_2_PtCl_4_, Na_2_PdCl_4_ and hydrochloric, (ii) Reduction using ascorbic acid at 35 °C for 4 h	
	M-Pd@Pt/NH_2_-GS	Mix and sonicate NH_2_-GS and M-Pd@Pt for 1 h	
Ir NPs-anti-CEA	PVP stabilized Ir NPs	(i) Add aqueous IrCl_3_ solution dropwise to ethanol solution containing PVP followed by mixing and stirring at 25 °C for 12 h, (ii) Refluxed in air at 100 °C for 6 h followed by evaporation	[79]
PBG-Au-anti-CEA; PPP-Au-anti-NSE; PTBO-Auanti-CA125; PMCP-Au-anti-Cyfra21–1; Cd NCs-Auanti-SCCA	PBG-Au	Add and stir TTAB to brilliant green aqueous solution followed by HAuCl_4_ addition and agitation for 4 h, RT	[80]
	PPP-Au	Add water with stirring to DMF solution of *N*-phenyl-p-phenylenediamine followed by HAuCl_4_ addition and agitation for 4 h, RT	
	PTBO-Au	Add HAuCl_4_ to toluidine blue o aqueous solution and agitate for about 4 h, RT	
	PMCP-Au	Add and stir DTAB to m-cresol purple ethanol solution followed by HAuCl_4_ addition and agitation for 4 h, RT	
	Cd NCs-Au	Mix Cd NCs with gold colloid and stirred for 4 h	
HRP-anti-CYFRA21-1/AuNPs/Thi/MWCNT-NH_2_	AuNPs	HAuCl_4_ reduction using NaBH_4_ in Thi/MWCNT-NH_2_ solution	[84]
	MWCNT-NH_2_	Acid treatment of MWCNT to get MWCNT-COOH followed by treatment with HMDA in presence of DCC for 96 h at 120 °C	
anti-AFP-Co_3_O_4_@MnO_2_-Thi	Co_3_O_4_@MnO_2_	Mixture of Co(CH_3_COO)_2_·4H_2_O and MnO_2_ nanotubes in ammonium hydroxide autoclaved at 150 °C, 5 h followed by calcination at 300 °C, 1 h	[85]
	Co_3_O_4_@MnO_2_-Thi	Co_3_O_4_@MnO_2_ treatment with APTES at 70 °C, 1.5 h followed by incubation with Thi at 95 °C, 1 h	
	anti-AFP-Co_3_O_4_@MnO_2_-Thi	EDC/NHS chemistry	
antiHER2/Hyd@AuNPs-APTMS-Fe_3_O_4_	antiHER2/Hyd@AuNPs-APTMS-Fe_3_O_4_	AuNPs preparation using HAuCl_4_ reduction via NaBH_4_, sodium citrate followed by treatment with APTMS-Fe_3_O_4_. Resulting AuNPs-APTMS-Fe_3_O_4_ were treated with thiolated anti-HER2 followed by treatment with hydrazine	[86]
Anti-CEA-AuNPs-Fc	AuNPs	Reduction of chloroauric acid with trisodium citrate	[87]
	Anti-CEA-AuNPs-Fc	Physical immobilization of anti-CEA on AuNPs followed by chemisorption of Fc-SH	
HRP-anti-CEA-AuNPs-TiO_2_-graphene	TiO_2_-graphene	Sonicate graphene with dopamine for 1 h, followed by stirring with TiO_2_	[89]
	HRP-anti-CEA-AuNPs-TiO_2_-graphene	Treat TiO_2_-graphene with HAuCl_4_ under ultraviolet irradiation followed by physical adsorption of HRP-anti-CEA	
PtCu@rGO/g-C_3_N_4_/anti-PSA	PtCu@rGO/g-C_3_N_4_/anti-PSA	Physical adsorption of anti-PSA on PtCu@rGO/g-C_3_N_4_	[90]
M-Pd@Pt/NH_2_-GS/anti-PSA	NH2-GS	GO prepared via modified Hummer’s method was mixed with ethylene glycol and ammonia followed by autoclaving at 180 °C for 10 h	[78]
	M-Pd@Pt	Pluronic F127 was mixed with K_2_PtCl_4_ and Na_2_PdCl_4_ in HCl followed by reducing with ascorbic acid	
Anti-AFP-Pt NPs/Co_3_O_4_/graphene	Pt NPs/Co_3_O_4_/graphene	Mix GO and Co(NO_3_)_2_·6H_2_O in ethanol and add ammonia solution followed by autoclaving at 190 °C for 24 h. Obtained Co_3_O_4_/graphene was mixed with Na_2_PtCl_4_ in ethanol aqueous solution and treat with NaBH_4_	[91]
GS-Fe_3_O_4_/Au@Ag/Ni^2+^-anti-CEA	NH2-GS-Fe_3_O_4_	GO prepared via modified Hummer’s method was mixed with clear solution of FeCl_3_·6H_2_O in ethylene glycol along with NaAc and ethanediamine and autoclaved at 200 °C for 8 h. Resulting GS-Fe_3_O_4_ was treated with APTES to get NH_2_-GS-Fe_3_O_4_	[92]
	Au@Ag	AuNPs prepared via citrate reduction were mixed with ascorbic acid, AgNO_3_ and CTAB solution and treated with NaOH	
	GS-Fe_3_O_4_/Au@Ag/Ni^2+^-anti-CEA	GS-Fe_3_O_4_/Au@Ag made by mixing NH2-GS-Fe_3_O_4_ and Au@Ag were dispersed in Ni(NO_3_)_2_·6H_2_O solution and stir for 24 h, anti CEA was immobilized via physical adsorption	
Ag NPs-MWCNTs/MnO_2_-Anti-CEA	Ag NPs-MWCNTs/MnO_2_	Acid treated MWCNTs were dispersed in KMnO_4_ solution and treated with MnSO_4_. Obtained MWCNTs/MnO_2_ were mixed with AgNO_3_ in water followed by reduction NaBH_4_	[93]
PdCu-anti-CEA	PdCu	Using AA as reducing agent and HDPC as growth inhibitor	[95]
anti-AFP-GNPs-HRP	GNP	Citrate reduction	[96]
anti-AFP/HRP-Au@ZnO	Au@ZnO	C_18_N_3_ was added to mixture of Zn(NO_3_)_2_ and HAuCl_4_ and heated at 145 °C, 5 h	[97]
anti-PSA/AuNPs	AuNPs	Citrate reduction in cold for smaller size and in boiling condition for large size	[98]
Co_3_O_4_@CeO_2_-Au@Pt-anti-SCCA	Co_3_O_4_@CeO_2_	Co(NO_3_)_2_·6H_2_O solution was treated with NaOH at 180 °C, 5 h. Obtained Co_3_O_4_ cubes were mixed in ethanol aqueous solution followed by addition of Ce(NO_3_)_3_ and HMT and refluxing at 70 °C, 2 h	[99]
	Au@Pt	Citrate reduced AuNPs were mixed with H_2_PtCl_6_ under boiling conditions followed by reduction with AA	
	Co_3_O_4_@CeO_2_-Au@Pt	APTES treated Co_3_O_4_@CeO_2_ were mixed with Au@Pt and stir for 12 h at room temperature.	
Anti-AFP/Au@Ag/PDA-PR-MCS	PR-MCS	C_6_H_5_OH and HCHO were added to solution containing NH_4_OH and C_2_H_5_OH and autoclaved at 100 °C, 24 h. Product was mixed with KOH and treated at 350 °C, 1 h followed by at 700 °C, 2 h	[100]
	Au@Ag	Citrate reduced AuNPs were mixed with AgNO_3_ solution and treated with NaBH_4_ solution	
	Au@Ag/PDA-PR-MCS	PR-MCS dispersed in tris buffer was treated with Dopamine hydrochloride 24 h and mixed with Au@Ag solution	
MSN-MB/PDA-anti-PSA	MSN	Mixture of CTAB and pluronics F127 in ethanol, water and ammonia was treated with TEOS	[101]
	MSN-MB/PDA	MB loaded MSN was treated with dopamine in tris buffer, pH 8.5	
Au@Pt DNs/NG/Cu^2+^-anti-CEA	NG	GO prepared via modified Hummer’s method was treated with ammonia solution at 90 °C, 4 h	[103]
	Au@Pt DNs	NaBH_4_ and AA reduced HAuCl_4_ and CTAB solution was mixed with K_2_PtCl_4_ and AA and treated at 60 °C, 12 h	
Au@Ag-Cu_2_O/anti-PSA	Au@Ag-Cu_2_O	Citrate reduced AuNPs were mixed with CTAC and AgNO_3_ followed by reduction using AA at 30 °C, 2 h. Obtained Au@Ag solution was mixed with CuCl_2_ and SDS followed by treatment with NaOH and NH_2_OH·HCl	[104]

Notes: AA: ascorbic acid; Apo: apoferritin; Cd NCs-Au: Cd nanocubes-gold; CdNCs and CuNCs: Cd_3_[Co(CN)_6_]_2_ and Cu_3_[Co(CN)_6_]_2_ nanocubes; CTAB: hexadecyl trimethyl ammonium bromide; CTAC: cetyltrimethylammonium chloride; HDPC: hexadecylpyridinium chloride monohydrate; HMDA: hexamethylenediamine; HMT: hexamethylenetetramine; Ir NPs: iridium nanoparticles; M-Alg: metal alginate nanobeads; MCF: mesoporous carbon form; MCM-41: multifunctional mesoporous silica; M-Pd@Pt/NH2-GS: mesoporous core-shell Pd@Pt nanoparticles loaded by amino group functionalized graphene; M-Pt NPs: mesoporous platinum nanoparticles; MSNs: mesoporous silica nanoparticles; N-MWCNTs: N-doped multi-walled carbon nanotubes; PBG-Au: poly (brilliant green)-gold; PDDA: poly(diallyldimethylammonium chloride); PMCP-Au: poly (m-cresol purple)-gold; PPP-Au: poly (*N*-phenyl-p-phenylenediamine)-gold; PTBO-Au: poly (toluidine blue o)-gold; PtPNPs: platinum porous nanoparticles; PVP: polyvinylpyrrolidone; SDS: sodium dodecyl sulfate.

**Table 3:** Characteristics of the developed immunoassays using electrochemical ELISA.

Probe	Immunosensor Conditions	Characteristics	Ref.
[DP]: anti-HER2-HRP [Anal]: HER2 [DM]: HQ	[Tran]: amperometry at −280 mV [IC]: (i) anal for 2 min at RT, (ii) [DP] for 20 min [MC]: 2.5 mm H_2_O_2_ with HQ in citrate buffer	[L]: 1 and 200 µg/mL, [LgS] [DL]: 1 µg/mL [S]: 18.23 µA/(µg/mL) [SL]: 3 weeks [CR]: 0.9591	[63]
[DP]: anti-CEA-HRP/AuNPs-PAN@CNTs [Anal]: CEA [DM]: H_2_O_2_	[Tran]: DPV in 0.2 to −0.8 V, [PA] 50 mV [IC]: (i) CEA, (ii) [DP] for 55 min at 37 °C, sequentially [MC]: 4 mM H_2_O_2_ in 5.0 mL PBS	[L]: (i) 0.02–3.0 ng/mL, (ii) 3.0–80 ng/mL [LS] [DL]: 0.008 ng/mL [S]: (i) 13.9465 µA/(ng/mL), (ii) 0.7342 µA/(ng/mL) [SL]: 30 days [CR]: (i) 0.9875, (ii) 0.9960	[47]
[DP]: AuNP–PAMAM dendrimer/PSA–aptamer-HRP [Anal]: PSA [DM]: thionine	[Tran]: DPV in −0.4 to −0.1 V [IC]: (i) PSA conc. for 15 min, (ii) [DP] for 20 min [MC]: 3 mM H_2_O_2_	[L]: 0.1 pg/mL to 90 ng/mL [LS] [DL]: 10 fg/mL [S]: 0.3635 µA/(pg/mL) [SL]: 3 weeks [CR]: 0.9831	[70]
[DP]: HRP-HRP-NP-hollow Au-NP-Thi@anti-AFP [Anal]: AFP [DM]: thionine	[Tran]: DPV in −0.4 to 0 V [IC]: (i) AFP conc for 16 min, 37 °C, (ii) [DP] for 30 min, RT [MC]: 4.2 mM H_2_O_2_	[L]: 0.025 to 5.0 ng/mL [LgS] [DL]: 8.3 pg/mL [S]: 7.649 µA/(ng/mL) [SL]: 30 days [CR]: 0.9949	[40]
[DP]: anti-AFP, HRP/MSNs-Fe_3_O_4_ [Anal]: AFP [DM]: thionine	[Tran]: CV in −0.6 to 0.6 V (vs. SCE) at 100 mV/s in PBS (pH 7.4) [IC]: (i) AFP, (ii) [DP] for 1 h, sequentially [MC]: 5 mmol/L H_2_O_2_ in PBS	[L]: 0.01 to 25 ng/mL [LS] [DL]: 4 pg/mL [SL]: 15 days	[64]
[DP]: GOx/anti-CEA/AgNPs [Anal]: CEA [DM]: H_2_O_2_	[Tran]: DPV in −0.2 to −0.8 V, [PA]: 50 mV, [PW]: 20 ms [IC]: (i) CEA conc 40 min, RT, (ii) [DP] 1 h, 4 °C [MC]: PBS + 1% glucose	[L]: 1 pg/mL to 50 ng/mL [LgS] [DL]: 0.27 pg/mL [S]: 8.281 µA/(ng/mL) [SL]: 30 days [CR]: 0.9971	[56]
[DP]: HRP-Ag@BSA-anti-CEA [Anal]: CEA [DM]: tyramine	[Tran]: DPV in 0 to -600 mV vs. SCE [PA]: 50mV, [PW]: 50 ms in PBS [IC]: (i) CEA conc 40 min, RT, (ii) [DP] 40 min, RT, (iii) 2 mM H_2_O_2_ + HRP-tyramine conjugates 10 min at RT [MC]: 2.5 mM H_2_O_2_ in PBS	[L]: 0.005–80 ng/mL [LgS] [DL]: 5.0 pg/mL [S]: 1.617 µA/(ng/mL) [SL]: 28 days [CR]: 0.9867	[108]
[DP]: HRP/HRP-anti-CA 19-9/Au@SBA-15 [Anal]: CA 19-9 [DM]: H_2_O_2_	[Tran]: chronoamperometry PBS pH 6 at −0.2 V [IC]: (i) CA 19-9 conc 1 h, 37 °C; (ii) [DP] 1 h, 37 °C [MC]: 3 mM H_2_O_2_ in PBS	[L]: 0.05 to 15.65 U/mL [LgS] [DL]: 0.01 U/mL [S]: 20.51 g/L [SL]: 30 days [CR]: 0.992	[74]
[DP]: anti-CEA –Ag/Au–DN-graphene [Anal]: CEA [DM]: Ag	[Tran]: CV in −0.6 to1.0 V (vs. SCE) at 50 mV/s [IC]: (i) CEA conc 30 min, (ii) [DP]: 40 min [MC]: 0.1M PBS (pH7.0).	[L]: 10 to 1.2 × 105 pg/mL [LgS] [DL]: 8 pg/mL [S]: 0.494 µA/(ng/mL) [CR]: 0.9899	[34]
[DP]: PAMAM-Gr/anti-AFP-HRP [Anal]: AFP [DM]: hydroquinone	[Tran]: (i) amperometric at −0.2 V, (ii) CV −0.5 to +0.5 V, 50 mV/s [IC]: (i) AFP conc 40 min, 37 °C, (ii) [DP]: 40 min, 37 °C [MC]: PBS containing 1 mM hydroquinone + 2 mM H_2_O_2_	[L]: 1.0–100 ng/mL [LS] [DL]: 0.45 ng/mL	[50]
[DP]: Au@Pd-Gra/Thi-anti-CA 19-9/HRP [Anal]: CA19-9 [DM]: Thionine	[Tran]: DPV in −0.4–0 V, [PA]: 50 mV, [PW]: 50 ms, [PP]: 0.2 s [IC]: (i) CA 19-9 conc 40 min, 25 °C, (ii) [DP] [MC]: 1.5 mM H_2_O_2_	[L]: 0.015 to 150 U/mL [LgS] [DL]: 0.006 U/mL [S]: 9.8328 µA/(U/mL) [SL]: 30 days [CR]: 0.9982	[72]
[DP]: HRP, GOD, anti-AFP/SWCNHs [Anal]: AFP [DM]: 4-CN	[Tran]: Impedance [IC]: (i) AFP conc 40 min, 37 °C, (ii) [DP] 40 min, 37 °C, (iii) 1.0 mM 4-CN and 10.0 mM glucose in 10mM PBS 15 min, RT [MC]: 0.01M PBS (pH 7.4) containing 5 mM FeCN_6_^3−/4−^ and 0.1M KCl	[L]: 0.001 to 60 ng/mL [LgS] [DL]: 0.33 pg/mL [S]: 230.60 Ω/(ng/mL) [SL]: 30 days [CR]: 0.996	[73]
[DP]: anti-CEA/Au/MCF [Anal]: CEA [DM]: Ag	[Tran]: ASV in −0.08 to 0.2 V, 50 mV/s [IC]: (i) CEA conc 40 min, 37 °C, (ii) [DP] 40 min, 37 °C, (iii) silver-deposition with enhancer solutions 4 min, 37 °C, [MC]: 1.0 M KCl	[L]: 0.05 pg/mL to 1 ng/mL [LgS] [DL]: 0.024 pg/mL [SL]: 15 days [CR]: 0.9997	[71]
[DP]: anti-AFP/CHIT–PB–AuNP; anti-CEA/CHIT–Fc–AuNPs, [Anal]: CEA, AFP [DM]: PB, Fc	[Tran]: DPV in −0.2 to 0.8 V [IC]: 45 min incubation for CEA, AFP concentration [MC]: PBS	[L]: 0.05–100 ng mL^−1^ for AFP and CEA [LgS] [DL]: 0.03 ng mL^−1^ and 0.02 ng mL^−1^ for AFP and CEA [S]: 0.47067 µA/(ng/mL), 0.51106 µA/(ng/mL) for AFP and CEA [CR]: 0.99712 for AFP and 0.99806 for CEA	[52]
[DP]: anti-AFP-AuNPs-Thi@rGO and anti-CEA-AuNPs-PB@rGO [Anal]: CEA, AFP [DM]: PB, Thionine	[Tran]: DPV 600 to −600 mV; [PA] 50 mV. [IC]: (i) CEA/AFP conc 50 min, 37 °C, (ii) [DP] 50 min, 37 °C [MC]: PBS pH 6.5	[L]: 0.6–80 ng/mL for both [LS] [DL]: 0.12 ng/mL and 0.08 ng/mL for CEA and AFP [S]: 0.0188 µA/(ng/mL), 0.0273 µA/(ng/mL) for CEA and AFP [SL]: 30 days [CR]: 0.9908, 0.9936 for CEA and AFP	[36]
[DP]: Anti-CEA/PB–CS-Au and anti APF/Cd–CS-Au [Anal]: CEA, AFP [DM]: PB, Cd	[Tran]: DPV in −0.1V to 0.9V (vs. Ag/AgCl), [PA]: 50 mV, [PW]: 50 ms [IC]: (i) CEA/AFP conc 40 min, (ii) [DP] mixture 1:1 [MC]: 0.1 M pH 6.5 phosphate buffered solution (PBS)	[L]: 0.01 to 100 ng/mL range for both [LgS] [DL]: 0.006 ng/mL for AFP and 0.01 ng/mL for CEA [S]: 1.771 µA/(ng/mL), 1.751 µA/(ng/mL) for CEA and AFP [CR]: 0.996 and 0.995 for CEA and AFP	[44]
[DP]: PLL-Au-Cd-Apo-anti-AFP and PLL-Au-Pb-Apo-anti-CEA [Anal]: AFP and CEA [DM]: Cd, PB	[Tran]: SWV scan from −1.0 to −0.3 V with frequency of 15 Hz, [PA]: 25 mV, potential step 4 mV, quiet time 2 s to measure AFP and CEA at −0.78 V and −0.53 V [IC]: (i) CEA/AFP conc 20 min, RT, (ii) [DP] 20 min, RT [MC]: (i) immuno-complex in acetate buffer containing 400 µg/L bismuth, (ii) deposition of bismuth film and metal ions in situ at −1.2 V for 120 s	[L]: 0.01–50 ng/mL for both [LgS] [DL]: 4 pg/mL for both [S]: 6.65 µA/(ng/mL), 6.62 µA/(ng/mL), for AFP and CEA [SL]: 25 days [CR]:0.992, 0.994 for AFP and CEA	[59]
[DP]: anti-CEA-PtPNP-Cd^2+^ and anti-AFP-PtPNPs-Cu^2+^ [Anal]: CEA and AFP [DM]:Cd^2+^, Cu^2+^	[Tran]: DPV in 0.2 to −0.9 V with [PA]: 50 mV, [PW]: 50 ms and quiet time of 2 s were recorded for CEA and AFP at −0.736 V and 0.004 V respectively [IC]: (i) CEA/AFP conc 1 h, 37 °C, (ii) [DP] 1 h, 37 °C [MC]: acetate buffer solution (0.2 M, pH 4.5).	[L]: 0.05 ng/mL to 200 ng/mL range for both CEA and AFP [LgS] [DL]: 0.002 ng/mL and 0.05 ng/mL for CEA and AFP [S]: 2.26 µA/(ng/mL), 1.06 µA/(ng/mL), for CEA and AFP [CR]: 0.997, 0.998 for CEA and AFP	[29]
[DP]: CdNCs–Au–anti-CEA and CuNCs–Au– anti-AFP [Anal]: CEA and AFP [DM]: CdNCs and CuNCs	[Tran]: SWV in 0.1 to −0.9 V with [PA]: 25 mV, pulse frequency 15 Hz, were recorded for CEA and AFP at −0.7 V and −0.1 V (vs. Ag/AgCl), [IC]: (i) CEA/AFP conc 50 min, 37 °C, (ii) [DP] 50 min, 37 °C [MC]: acetate buffer solution (0.2 M, pH 6).	[L]: 0.025 to 250 ng/mL range for both [LgS] [DL]: 0.0175 ng/mL and 0.0109 ng/mL for CEA and AFP [S]: 4.31 µA/(ng/mL), 3.858 µA/(ng/mL), for CEA and AFP [CR]: 0.998 for CEA and AFP	[53]
[DP]: TB/Au@KIT-6/CMC/ILs-anti-CEA [Anal]: CEA [DM]: TB	[Tran]: DPV in −0.6 V to 0 V [IC]: (i) CEA conc 1 h, RT, (ii) [DP] 1 h [MC]: PBS pH 6.8	[L]: 10−5 ng/mL to 102 ng/mL [LgS] [DL]: 3.3 fg/mL [S]: 3.32 µA/(ng/mL) [SL]: 2 weeks [CR]: 0.99	[45]
[DP]: Cd-Alg-anti-AFP, Pb-Alg-anti-CEA and Cu-Alg-anti-PSA [Anal]: AFP, CEA and PSA [DM]: Cd, Pb, Cu	[Tran]: DPV in −0.9 to 0.2 V to measure AFP, CEA and PSA at −0.76 V, −0.5 V and 0.12 V (vs. Ag/AgCl) [IC]: (i) CEA/AFP/PSA conc 50 min, 37 °C, (ii) [DP] 50 min, 37 °C [MC]: acetate buffer solution (0.2 M, pH 5).	[L]: 0.01 to 100 ng mL^−1^ for all [DL]: 0.01, 0.0086 and 0.0075 ng/mL for AFP, CEA and PSA [S]: 5.548 µA/(ng/mL), 3.737 µA/(ng/mL), 4.586 µA/(ng/mL), for AFP, CEA and PSA [SL]: 15 days [CR]: 0.993, 0.994, 0.996 for AFP, CEA and PSA	[43]
[DP]: anti-CA19-92/Envision/Au, anti-AFP2/Envision/CdS and anti-CEA2/Envision/PbS [Anal]: CA19-9, CEA and AFP [DM]: Au (via CSV), CdS, PbS (via ASV)	[Tran]: ASV with accumulation at −1.2 V for 120 s, and scanning from −1.0 to −0.3 V, with [PS]: 4 mV, frequency 15 Hz, and [PA]: 25 mV. CSV +1.3 V for 30 s, immediately followed by DPV detection from +0.6 V to 0 V, with [PS]: 4 mV, [PA]: 50 mV, and pulse period of 0.2 s. [IC]: (i) Ca 19-9/CEA/AFP conc 30 min, RT, (ii) [DP] 30 min, RT [MC]: (i) GCE was incubated in pH 2.0 bismuth nitrate solution in acetate and treated at −1.2 V for 120 s, (ii) immune-complex in 0.1 M HCl	[L]: 5 pg/mL–100 ng/mL, 1 pg/mL–50 ng/mL, and 1 pg/mL–50 ng/mL for CA19-9, CEA and AFP [LgS] [DL]: 0.3, 0.05, 0.02 pg/mL for CA19-9, CEA and AFP [S]: 6.65, 7.32, 0.60 µA/(ng/mL) for AFP , CEA and CA19-9 [SL]: 60 days [CR]:0.99, 0.997, 0.993 for AFP , CEA and CA19-9	[107]
[DP]: Au@MCM-41/TB/anti-AFP [Anal]: AFP [DM]: TB	[Tran]: DPV in −0.6 V to 0.2 V. [IC]: (i) AFP conc 1 h, RT, (ii) [DP] 1 h, RT [MC]: PBS pH 6.8	[L]: 10−4 ng/mL to 103 ng/mL [LgS] [DL]: 0.05 pg/mL [S]: 1.43 µA/(ng/mL), [SL]: 2 weeks [CR]: 0.99	[57]
[DP]: Au@CMK-3-anti-CEA-neutral red and Au@CMK-3-anti-SCCA-thionine [Anal]: CEA and SCCA [DM]: neutral red, thionine	[Tran]: DPV in −0.7 to 1 V for recording −0.62 V (neutral red), and −0.17V (thionine) [IC]: (i) CEA/SCCA conc 1 h, RT, (ii) [DP] 1 h [MC]: PBS pH 7.4	[L]: 0.05 to 20 ng/mL and 0.03 to 20 ng/mL range for CEA and SCCA [LS] [DL]: 0.013 ng/mL and 0.010 ng/mL for CEA and SCCA [SL]: 10 days	[66]
[DP]: AuNPs–anti-CEA–Cu^2+^ and AuNPs–anti-AFP–Pb^2+^ [Anal]: CEA and AFP [DM]: Cu^2+^, Pb^2+^	[Tran]: DPV in −0.7 V to 0.3 V (vs. SCE), [PA] 50 mV, [PW] 50 ms [IC]: (i) CEA/AFP conc 35 min, 37 °C, (ii) [DP] 45 min, 37 °C [MC]: HAc/NaAc (0.2 M, pH 3.5)	[L]: 0.01–50 ng/mL for both [LgS] [DL]: 4.6 pg/mL and 3.1 pg/mL for CEA and AFP [S]: 3.3 µA/(ng/mL), 4.86 µA/(ng/mL), for CEA and AFP [CR]:0.9967, 0.9991 for CEA and AFP	[54]
[DP]: Aq-SA/bio-dsDNA/SA/bio-anti-AFP/Au/SiO_2_–Fe_3_O_4_, Thi-SA/bio-dsDNA/SA/bio-anti-CEA/Au/SiO_2_–Fe3O_4_, Co-SA/bio-dsDNA/SA/bio-anti-CA125/Au/SiO_2_–Fe_3_O_4_, Fc-SA/bio-dsDNA/SA/bio-anti-PSA/Au/SiO_2_–Fe_3_O_4_ [Anal]: AFP, CEA, CA125 and PSA [DM]: Aq, Thi, Co, Fc	[Tran]: DPV in −0.7 to 0.7 V incre: 0.004 V, [PA]: 0.05 V, [PW]: 0.05 s, sampling width: 0.0167 s, pulse period: 0.2 s to record AFP at −0.52 V, CEA at −0.21V, CA125 at 0.0V, PSA at 0.26V [IC]: (i) AFP/CEA/CA125/PSA conc 40 min, 37 °C, (ii) [DP]: 40 min, 37 °C [MC]: PBS 0.1 M, pH: 7.4	[L]: 0.2 to 800 pg/mL, 0.2 to 600 pg/mL, 0.2 to 1000 pg/mL, and 0.2 to 800 pg/mL for AFP, CEA, CA125 and PSA [LgS] [DL]: 62, 48, 77 and 60 fg/mL for AFP, CEA, CA125 and PSA [S]: 22.71 µA/(pg/mL), 21.91 µA/(pg/mL), 33.69 µA/(pg/mL),21.30 µA/(pg/mL), for for AFP, CEA, CA125 and PSA [SL]: 14 days [CR]: 0.9797, 0.9696, 0.9791, 0.9786 for AFP, CEA, CA125 and PSA	[46]
[DP]: anti-CEA-AuNP-So [Anal]: CEA [DM]: MB	[Tran]: DPV 0 to −500 mV vs SCE [IC]: (i) CEA conc + [DP] 40 min, RT, (ii) hybridization mix 80 min, RT, (iii) hemin incubation 50 min RT, (iv) methylene blue incubation 30 min at RT [MC]: PBS (pH 7.0) containing 3.0 mM H_2_O_2_	[L]: 1.0 fg/mL to 20 ng/mL [LgS] [DL]: 0.5 fg/mL [S]: 1.9636 µA/(ng/mL), [SL]: 30 days [CR]: 0.9973	[60]
[DP]: anti-CA15-3–f-TiO_2_–Cd^2+^ [Anal]: CA15-3 [DM]: Cd^2+^	[Tran]: SWV in −1 to −0.45 V [IC]: (i) CA15-3 conc 1 h, 4 °C, (ii) [DP] 1 h, 4 °C [MC]: PBS (pH 5.4)	[L]: 0.02–60 U/mL [LS] [DL]: 0.008 U/mL [S]: 1.806 µA/(U/mL), [SL]: 4 weeks [CR]: 0.998	[32]
[DP]: PtNP@ICP-anti-PSA [Anal]: PSA [DM]: FcDA	[Tran]: DPV at 0.31 V vs. SCE [IC]: (i) PSA conc 30 min, RT, (ii) [DP] 30 min, RT, [MC]: 5.0 mM H_2_O_2_ in the PBS pH 7.0	[L]: 0.001 to 60 ng/mL [LgS] [DL]: 0.3 pg/mL [S]: 1.85129 µA/(ng/mL), [SL]: 1 week [CR]: 0.988	[61]
[DP]: anti-PSA-Fc-AuNP [Anal]: PSA [DM]: Fc	[Tran]: DPV in 0 to 0.6 V [IC]: (i) PSA conc 75 min, 37 °C, (ii) [DP] 90 min, 37 °C, [MC]: PBS	[L]: 10 pg/mL to 100 ng/mL [LS] [DL]: 5.4 pg/mL [S]: 0.137 µA/(ng/mL), [CR]: 0.9907	[68]
[DP]: Aptamer/Thi-AuNPs/SiO_2_@MWCNTs [Anal]: MUC 1 [DM]: Thionine	[Tran]: DPV in −0.33 to −0.1 V [IC]: (i) MUC1 conc 40 min, 37 °C, (ii) [DP] 40 min, 37 °C, [MC]: PBS pH 7.4	[L]: 10^−3^ to 1 nM, 1–100 nM [LS] [DL]: 1 pM [S]: 1.647 nA/nM [SL]: 30 days [CR]: 0.98	[58]
[DP]: HRP-GOD/Fc-anti-AFP/PGN, HRP-GOD/Thi-anti-CEA/PGN [Anal]: CEA and AFP [DM]: Fc, Thi	[Tran]: SWV in −0.6 V to 0.6 V with a frequency of 15 Hz and a [PA]: of 25 mV (vs. SCE) to record Thi (at −0.15 V) and Fc (at 0.35 V) [IC]: (i) CEA/AFP conc 45 min, 37 °C, (ii) [DP] 45 min, 37 °C, [MC]: PBS (0.1 M pH 6.5) with 4 mM glucose	[L]: 0.01–100 ng/mL for both [LgS] [DL]: 1.64 pg/mL and 1.33 pg/mL for CEA and AFP [SL]: 30 days [CR]: 0.998, 0.994 for CEA and AFP	[48]
[DP]: 3DGS@MB-anti-CEA and 3DGS@Fc-anti-AFP [Anal]: CEA and AFP [DM]: MB, Fc	[Tran]: DPV in −0.4 to 0.4 V with [PA]: 50 mV and [PW] 50 ms [IC]: (i) CEA/AFP conc 40 min, RT, (ii) [DP], [MC]: PBS (pH 7.0, containing 0.1 M KCl)	[L]: 0.001 to 100 ng/mL for both [LgS] [DL]: 0.5 and 0.8 pg/mL for CEA and AFP [S]: 11.19, 27.866 µA/(ng/mL), for CEA and AFP [SL]: 10 days [CR]: 0.9985, 0.9957 for CEA and AFP	[76]
[DP]: CGN-Thi-anti-CEA, CGN-DAP-anti-PSA and CGN-Cd^2+^-anti-AFP [Anal]: PSA, CEA, AFP [DM]: DAP, Thi, Cd^2+^	[Tran]: SWV −1.2 V to 0.2 V (vs. SCE) [PA] 50 mV [PW] 50 ms to record Thi, DAP and Cd^2+^ at −0.05 V, −0.35 V and −0.65 V [IC]: (i) PSA/CEA/AFP conc 35 min, 37 °C, (ii) [DP] 45 min, 37 °C, [MC]: PBS (pH 6.5, 0.1 M)	[L]: 0.01–100 ng/mL for all three [LgS] [DL]: 4.8, 2.7 and 3.1 pg/mL for PSA, CEA and AFP [S]: 4.12, 5.84, 5.48 µA/(ng/mL), for PSA, CEA and AFP [SL]: 2 weeks [CR]: 0.997, 0.995, 0.997 for PSA, CEA and AFP	[75]
[DP]: M-Pt-anti-CA125, M-Pt-anti-CA153, M-Pt-anti-CEA [Anal]: CEA, CA153, CA125	[Tran]: DPV in −0.65 to 0.4 V [IC]: (i) CEA, CA153, CA125 conc 1 h, RT, (ii) [DP] 1 h, RT [MC]: PBS (pH 7.4) containing 5 mM H_2_O_2_	[L]: 0.02–20 ng/mL, 0.008–24 U/mL, 0.05–20 U/mL for CEA, CA153 and CA125, [LgS] [DL]: 7.0 pg/mL, 0.001 U/mL and 0.002 U/mL, for CEA, CA153 and CA125 [SL]: one month [CR]: 0.9927, 0.9962, 0.9988 for CEA, CA153 and CA125	[30]
[DP]: anti-CA72-4/PANi–Au AMNPs [Anal]: CA72-4 [DM]: H_2_O_2_	[Tran]: amperometric at −0.4 V [IC]: (i) CA72-4 conc 1 h, RT, (ii) [DP] 1 h, RT [MC]: PBS pH 7.4 with 5.0 mmol/L H_2_O_2_	[L]: 2 to 200 U/mL [LS] [DL]: 0.10 U/mL [S]: 0.814 µA/(U/ml) [SL]: 20 days [CR]: 0.9945	[55]
[DP]: Fe_3_O_4_@SiO_2_–Fc–anti-CEA/HRP [Anal]: CEA [DM]: Fc	[Tran]: DPV in −0.1 to 0.8 V [IC]: (i) CEA conc 40 min, RT, (ii) [DP] [MC]: PBS (pH 7.4) with 4 mM H_2_O_2_	[L]: 0.001 to 80 ng/mL [LS] [DL]: 0.0002 ng/mL [S]: 0.3867 µA/(ng/mL) [SL]: 3 weeks [CR]: 0.99	[35]
[DP]: anti-SCCA/Pd–Au/C [Anal]: SCCA [DM]: H_2_O_2_	[Tran]: amperometric at −0.2 V [IC]: (i) SCCA conc, (ii) [DP] 1 h [MC]: PBS pH 6.8 with H_2_O_2_	[L]: 0.005 to 2 ng/mL [LS] [DL]: 1.7 pg/mL [S]: 4.351 µA/(ng/mL) [SL]: 7 days [CR]: 0.9995	[28]
[DP]: Cu@Ag-CD-ADA-anti-CEA [Anal]: CEA [DM]: H_2_O_2_	[Tran]: amperometric at −0.4 V [IC]: (i) CEA conc, 60 min, RT, (ii) [DP] 60 min, RT [MC]: PBS pH 7.0 with 5mM H_2_O_2_	[L]: 0.0001–20 ng/mL [LS] [DL]: 20 fg/mL [S]: (i) 212.46 µA/(ng/mL) below 0.5 ng/mL, (ii) 5.82 µA/(ng/mL) above 0.5 ng/mL [SL]: 1 week [CR]: (i) 0.9955, (ii) 0.9982	[37]
[DP]: anti-AFP/Fe_3_O_4_@C@Pd [Anal]: AFP [DM]: H_2_O_2_	[Tran]: amperometric at −0.4 V [IC]: (i) AFP conc, 1 h, (ii) [DP] [MC]: PBS pH 6.5 with 5 mM H_2_O_2_	[L]: 0.5 pg/mL to 10 ng/mL [LgS] [DL]: 0.16 pg/mL [S]: 45.195 µA/(ng/mL) [SL]: 30 days [CR]: 0.981	[33]
[DP]: anti-CEA/NP-PtFe [Anal]: CA15-3 [DM]: H_2_O_2_	[Tran]: chronoamperometry at −0.4V [IC]: (i) CA15-3 conc, 1 h, RT, (ii) [DP] 1 h, RT [MC]: PBS pH 7.4 with 5 mM H_2_O_2_	[L]: 0.002 to 40 U/mL [LS] [DL]: 3 × 10^−4^ U/mL [S]: 1.879 µA/(U/mL) [SL]: 10 days [CR]: 0.9988	[31]
[DP]: anti-AFP/PdNi/N-GNRs [Anal]: AFP [DM]: H_2_O_2_	[Tran]: DPV [IC]: (i) AFP conc, 1 h, RT, (ii) [DP] 1 h, RT [MC]: PBS pH 7.0 with 5 mM H_2_O_2_	[L]: 0.0001–16 ng/mL [LS] [DL]: 0.03 pg/mL [S]: (i) 161.86 µA/(ng/mL) below 0.2 ng/mL, (ii) 9.09 µA/(ng/mL) above 0.2 ng/mL [SL]: 20 days [CR]: (i) 0.9946, (ii) 0.9969	[39]
[DP]: Pb^2+^@Au@MWCNT-Fe_3_O_4_/anti-AFP [Anal]: AFP [DM]: H_2_O_2_	[Tran]: amperometric at −0.4 V [IC]: (i) AFP conc, 1 h; RT (ii) [DP] [MC]: PBS pH 7.4 with 5 mM H_2_O_2_	[L]: 10 fg/mL to 100 ng/mL [LgS] [DL]: 3.33 fg/mL [S]: 11.19 µA/(ng/mL) [SL]: 4 weeks [CR]: 0.9984	[49]
[DP]: anti-SCCA/Au/Ag/Au NPs [Anal]: SCCA [DM]: H_2_O_2_	[Tran]: amperometric at −0.4 V [IC]: (i) SCCA conc, 1 h, RT, (ii) [DP] 1 h, RT [MC]: PBS pH 7.17 with 5 mM H_2_O_2_	[L]: 0.5 pg/mL to 40 ng/mL [LgS] [DL]: 0.18 pg/mL [S]: 25.33 µA/(ng/mL) [SL]: 2 weeks [CR]: 0.9880	[41]
[DP]: anti-AFP/Pd/APTES-M-CeO_2_-GS [Anal]: AFP [DM]: H_2_O_2_	[Tran]: amperometric at −0.4 V [IC]: (i) AFP conc, 1 h, 4°C, (ii) [DP] 1 h, [MC]: PBS pH 7.4 with 5 mM H_2_O_2_	[L]: 0.1 pg/mL to 50 ng/mL [LgS] [DL]: 0.033 pg/mL [S]: 10.1 µA/(ng/mL) [SL]: 4 weeks [CR]: 0.99	[51]
[DP]: anti-SCC/Pt–Fe_3_O_4_ NPs [Anal]: SCC [DM]: H_2_O_2_	[Tran]: amperometric at −0.4 V [IC]: (i) SCC conc, 1 h, (ii) [DP] 1 h, [MC]: PBS pH 7.4 with 5 mM H_2_O_2_	[L]: 0.05 to 18 ng/mL [DL]: 15.3 pg/mL [SL]: 20 days	[65]
[DP]: anti-CA72-4/PtPd-Fe_3_O_4_ NPs [Anal]: CA72-4 [DM]: H_2_O_2_	[Tran]: amperometric at −0.4 V [IC]: (i) SCC conc, 1 h, 4 °C, (ii) [DP] 1 h, [MC]: PBS pH 7.0 with 5 mM H_2_O_2_	[L]: 0.001–10 U/mL [DL]: 0.0003 U/mL [SL]: 10 days	[67]
[DP]: CNTs/PDDA/HRP/ConA/HRP-anti-CEA [Anal]: CEA [DM]: hydroquinone	[Tran]: DPV in −0.4 to 0.2 V (vs. SCE) at a scan rate of 50 mV/s [IC]: (i) CEA conc, 40 min, RT, (ii) [DP] 60 min, RT [MC]: PBS (0.02 M, pH 7.5) with 2 mM H_2_O_2_ and 3 mM HQ	[L]: (i) 0.05–5 ng/mL and (ii) 5–200 ng/mL, [LS] [DL]: 0.018 ng/mL [S]: (i) 1.29 µA/(ng/mL), (ii) 0.0315 µA/(ng/mL) [SL]: 15 days [CR]: (i) 0.998, (ii) 0.998	[62]
[DP]: M-Pd@Pt/NH_2_-GS/anti-PSA [Anal]: PSA [DM]: H_2_O_2_	[Tran]: amperometric at −0.4 V [IC]: (i) PSA conc, 1 h, 4 °C, (ii) [DP] 40 min, RT, [MC]: PBS pH 7.38 with 5 mM H_2_O_2_	[L]: 10 fg/mL–50 ng/mL [LgS] [DL]: 3.3 fg/mL [S]: 11.96 µA/(ng/mL), [SL]: 4 weeks [CR]: 0.9988	[78]
[DP]: Ir NPs-anti-CEA [Anal]: CEA [DM]: H_2_O_2_	[Tran]: amperometric at −0.6 V [IC]: (i) CEA conc, (ii) [DP] 1 h, 37 °C, [MC]: PBS pH 7.4 with 5 mM H_2_O_2_	[L]: 0.5 pg/mL–5 ng/mL [LgS] [DL]: 0.23 pg/mL [S]: 0.435 µA/(ng/mL), [SL]: 30 days [CR]: 0.99	[79]
[DP]: PBG-Au-anti-CEA; PPP-Au-anti-NSE; PTBO-Au anti-CA125; PMCP-Au-anti-Cyfra21–1; Cd NCs-Au anti-SCCA [Anal]: CEA, NSE, CA125, Cyfra21–1, SCCA [DM]: PBG-Au, PPP-Au, PTBO-Au, PMCP-Au and Cd NCs at 0.4 V, 0.15 V, −0.14 V, −0.5 V, −0.75 V	[Tran]: SWV in −1.0 V to 0.8 V to record peaks at 0.4 V, 0.15 V, −0.14 V, −0.5 Vand −0.75 V (vs. Ag/AgCl) for simultaneously detection of CEA, NSE, CA125, Cyfra21–1 and SCCA [IC]: (i) CEA, SCCA, CA125, Cyfra21–1 and NSE mix, (ii) PBG-Au-anti-CEA, PPP-Au-anti-NSE, PTBO-Au-anti-CA125, PMCP-Au-anti-Cyfra21–1, Cd NCs-Au-anti-SCCA probes mixture 45 min, 37 °C. [MC]: PB (0.1 M, pH 6.0).	[L]: 0.1 to 100 ng/mL for SCCA, 1 to 150 ng/mL for CEA, NSE and Cyfra21–1, and 1 to 150 U/mL for CA125 [LgS] [DL]: 0.2 ng/mL for CEA, 0.9 ng/mL for NSE, 0.9 U/mL for CA125, 0.4 ng/mL for Cyfra21–1 and 0.03 ng/mL for SCCA [S]: 3.06 µA/(ng/mL), 4.9 µA/(ng/mL), 3.7 µA/(U/mL), 2.3 µA/(ng/mL), 2.57 µA/(ng/mL), for CEA, NSE, CA125, Cyfra21–1, SCCA [SL]: 4 weeks [CR]: 0.984, 0.983, 0.997, 0.995 and 0.971 for CEA, NSE, CA125, Cyfra21–1, SCCA	[80]
[DP]: HRP-MNP-anti-PSA, HRP-MNP-anti-PSMA, HRP-MNP-anti-IL-6, HRP-MNP-anti-PF-4 [Anal]: PSA, PSMA, IL-6, PF-4 [DM]: HQ	[Tran]: DPV from 0.0 V to −0.4 V vs. Ag/AgCl at 4 mV step, 25 mV amplitude, and 0.5 s pulse and 15 Hz [IC]: (i) [Anal]: mix with [DP], (ii) incubation with sensor [MC]: PBS with 1 mM HQ and 100 µM H_2_O_2_	[L]: 2 pg/mL to 200 ng/mL for PSA, 0.05 pg/mL to 5 ng/mL for IL-6, 0.1 pg/mL to 10 pg/mL for PF-4, and 0.15 pg/mL to 15 ng/mL for PSMA [LgS] [S]: 0.84 ± 0.06 nA/(pg/mL), for PSA, 0.90 ± 0.09 nA/(pg/mL), for IL-6, 0.98 ± 0.07 nA/(pg/mL), for PF-4, and 1.1 ± 0.1 nA/(pg/mL), for PSMA [SL]: 7 days	[81]
[DP]: AuNP-HRP, anti-PSA [Anal]: PSA [DM]: TMB	[Tran]: amperometric at −0.1 V [IC]: (i) PSA conc 250 µL at 30 µL/min flow, (ii) [DP] [MC]: PBS pH 7.4 with TMB-H_2_O_2_	[L]: 0.2–12.5 ng/mL [LS] [DL]: 0.2 ng/mL [S]: 2.24 nA/(ng/mL) [CR]: 0.94	[82]
[DP]: Primer-AuNP-PSA aptamer [Anal]: PSA [DM]: CuNPs	[Tran]: DPSV in −0.2 to 0.6 V with 4 mV step, amplitude 0.05 V, [PW]: 0.05 s, pulse period 0.5 s, deposition potential −0.5 V, deposition time 300 s [IC]: (i) PSA conc, 1 h, 37 °C, (ii) [DP] 1 h, 37 °C, (iii) RCA reaction, (iv) CuNP formation, 30 min, RT [MC]: Cu^2+^ in 0.5 M HNO_3_	[L]: 0.05–500 fg/mL [LgS] [DL]: 0.02 fg/mL [S]: 3.48 µA/(fg/mL) [CR]: 0.995	[83]
[DP]: anti-CYFRA-1-HRP/AuNPs/Thi/MWCNT-NH_2_ [Anal]: CYFRA-1 [DM]: Thi	[Tran]: DPV in −0.4 to 0 V [IC]: (i) CYFRA-1 conc for 1 h, 35 °C (ii) [DP] for 1 h, 35 °C [MC]: 2 mM H_2_O_2_	[L]: 0.1–150 ng/mL [LS] [DL]: 43 pg/mL [S]: 0.446 µA/(ng/mL) [SL]: 15 days [CR]: 0.9937	[84]
[DP]: anti-AFP-Co_3_O_4_@MnO_2_-Thi [Anal]: AFP [DM]: Thi	[Tran]: DPV in −0.6 to 0.6 V [IC]: (i) AFP conc, (ii) [DP] [MC]: dried 10 µL unbound [DP] on AgNP/SPEC using 50 µL PBS	[L]: 0.001–100 ng/mL [LgS] [DL]: 0.33 pg/mL [S]: 5.24 µA/(ng/mL) [CR]: 0.9977	[85]
[DP]: antiHER2/Hyd@AuNP-APTMS-Fe_3_O_4_ [Anal]: HER2 [DM]: AgNPs	[Tran]: DPV in 0 to 0.6 V [IC]: (i) HER2 conc for 30 min, 37 °C, (ii) [DP] 30 min, 37 °C, 0.01 M AgNO_3_, 25 min [MC]: 0.01 M AgNO_3_, 25 min	[L]: 5 × 10^−4^ to 50.0 ng/mL [LgS] [DL]: 2.0 × 10−5 ng/mL [S]: 1.9194 µA/(ng/mL) [CR]: 0.9906	[86]
[DP]: anti-CEA-AuNP-Fc [Anal]: CEA [DM]: Fc	[Tran]: SWV in 0 to 0.6 V [IC]: (i) CEA conc for 45 min, (ii) [DP] for 45 min [MC]: 0.1 M PBS (pH~7.0)	[L]: 0.5 to 10 ng/mL [LS] [DL]: 0.2 ng/mL [S]: 0.4494 µA/(ng/mL) [SL]: 3 weeks [CR]: 0.9968	[87]
[DP]: anti-PSA-HRP [Anal]: PSA [DM]: MB	[Tran]: SWV in −0.4 to 0.15 V [IC]: (i) PSA conc for 25 min, (ii) [DP] for 30 min, [MC]: 1 mM MB + 2.5 mM H_2_O_2_	[L]: 1–18 ng/mL [LS] [DL]: 1 pg/mL [S]: 3.234 µA/(ng/mL) [SL]: 3 weeks [CR]: 0.996	[88]
[DP]: HRP-anti-CEA-AuNP-TiO_2_-GR [Anal]: CEA [DM]: HQ	[Tran]: DPV in 0.55 to −0.3 V [IC]: (i) CEA conc for 30 min, (ii) [DP] for 1 h, 35 °C [MC]: 2 mM H_2_O_2_ + 2.5 mM HQ	[L]: 0.005–200 ng/mL [LgS] [DL]: 3.33 pg/mL [S]: 11.98 µA/(ng/mL) [SL]: 15 days [CR]: 0.994	[89]
[DP]: PtCu@rGO/g-C_3_N_4_/anti-PSA [Anal]: PSA [DM]: Thi	[Tran]: amperometric −0.4 V [IC]: (i) PSA conc for 30 min, (ii) [DP] for 50 min, RT [MC]: 5 mM H_2_O_2_	[L]: 50 fg/mL to 40 ng/mL [LgS] [DL]: 16.6 fg/mL [S]: 15.97 µA/(ng/mL) [SL]: 4 weeks [CR]: 0.9913	[90]
[DP]: M-Pd@Pt/NH_2_-GS/anti-PSA [Anal]: PSA [DM]: H_2_O_2_	[Tran]: amperometric −0.4 V [IC]: (i) PSA conc for 1 h, 4°C, (ii) [DP] for 40 min, RT [MC]: 5 mM H_2_O_2_	[L]: 10 fg/mL to 50 ng/mL [LgS] [DL]: 3.3 fg/mL [S]: 11.96 µA/(ng/mL) [SL]: 4 weeks [CR]: 0.9988	[78]
[DP]: anti-AFP-Pt NPs/Co_3_O_4_/graphene [Anal]: AFP [DM]: H_2_O_2_	[Tran]: amperometric −0.4 V [IC]: (i) AFP conc for 1 h, 4 °C, (ii) [DP] for 1 h, 4 °C [MC]: 5 mM H_2_O_2_	[L]: 0.1 pg m/L to 60 ng/mL [LgS] [DL]: 0.029 pg/mL [S]: 9.71 µA/(ng/mL) [SL]: 28 days [CR]: 0.996	[91]
[DP]: GS-Fe_3_O_4_/Au@Ag/Ni^2+^-anti-CEA [Anal]: CEA [DM]: H_2_O_2_	[Tran]: amperometric −0.4 V [IC]: (i) CEA conc for 1 h, RT, (ii) [DP] for 1 h, RT [MC]: 5 mM H_2_O_2_	[L]: 0.1 pg/mL to 100 ng/mL [LgS] [DL]: 0.0697 pg/mL [S]: 6.62 µA/(ng/mL) [SL]: 2 weeks [CR]: 0.998	[92]
[DP]: HRP-anti-CEA [Anal]: CEA [DM]: Thi	[Tran]: DPV in −0.4 to 0.1V [IC]: (i) CEA conc + [DP] for 30 min, RT [MC]: 0.5 mM Thi + 5 mM H_2_O_2_in PBS	[L]: 0.02 to 12 ng/mL [LS] [DL]: 0.01 ng/mL [SL]: 4 weeks [CR]: 0.998	[94]
[DP]: PdCu-anti-CEA [Anal]: CEA [DM]: polyaniline	[Tran]: DPV in −0.2 to 0.6 V [IC]: (i) CEA conc for 1 h, (ii) [DP] RT [MC]: aniline polymerization by CV in −1 to 1 V, 200 s, 100 mV/s	[L]: 0.1 pg/mL to 10.0 ng/mL [LgS] [DL]: 0.08 pg/mL [S]: 2.49 µA/(ng/mL) [SL]: 5 weeks [CR]: 0.99	[95]
[DP]: anti-AFP-GNPs-HRP [Anal]: AFP [DM]: HQ	[Tran]: amperometric −0.2 V [IC]: (i) AFP conc + [DP] for 20 min, RT [MC]: 2mM HQ + 2 mM H_2_O_2_	[L]: 20 to 100 ng/mL [LS] [DL]: 0.64 ng/mL [S]: 0.3869 µA/(ng/mL) [SL]: 15 days [CR]: 0.9940	[96]
[DP]: streptavidin-HRP [Anal]: HER2 [DM]: TMB	[Tran]: CV in −0.2 to 0.8 V, 50 mV/s [IC]: (i) HER2 conc for 2 h, RT and dried, (ii) [DP] for 2 h, RT and dried, (iii) streptavidin-HRP 30 min, RT, (iv) TMB-H_2_O_2_ 20 min	[L]: 5 to 20 ng/mL and 20 to 200 ng/mL [LS] [DL]: 4 ng/mL and 5 ng/mL [S]: 0.087 µA/(ng/mL) and 0.28 µA/(ng/mL) [SL]: 7 days	[109]
[DP]: anti-AFP/HRP-Au@ZnO [Anal]: AFP [DM]: TMB	[Tran]: DPV in −0.1 to 0.6 V [IC]: (i) AFP conc for 40 min, 37 °C, (ii) [DP] [MC]: 0.1 mM TMB + 0.2 mM H_2_O_2_	[L]: 0.02 pg/mL to 10ng/mL and 10 to 100 ng/mL [LgS] [DL]: 0.01 pg/mL [S]: 1.48 µA/(10^−10^ g/mL) and 6.15 µA/(10^−10^ g/mL) [SL]: 1 week [CR]: 0.9956 and 0.9917	[97]
[DP]: anti-PSA/AuNPs [Anal]: PSA [DM]: Ag (I) ions	[Tran]: Linear sweep anodic striping in −0.2 to 0.5 V, 50 mV/s [IC]: (i) PSA conc for 30 min, RT, (ii) [DP] for 40 min, RT, (iii) 1st Au enhancement, (iv) spiky gold enhancement, (v) silver enhancement [MC]: 1 M KCl	[L]: 1.95 to 125 pg/mL and 0.125 to 10 ng/mL [LS] [DL]: 1.2 pg/mL [S]: 17.51 µA/(ng/mL) and 3.5 µA/(ng/mL) [CR]: 0.99 and 0.9896	[98]
[DP]: Co_3_O_4_@CeO_2_-Au@Pt-anti-SCCA [Anal]: SCCA [DM]: H_2_O_2_	[Tran]: amperometric −0.4 V [IC]: (i) SCCA conc for 1 h, (ii) [DP] for 1 h [MC]: 5 mM H_2_O_2_	[L]: 100 fg/mL to 80 ng/mL [LgS] [DL]: 33 fg/mL [S]: 4.43 µA/(ng/mL) [SL]: 4 weeks [CR]: 0.998	[99]
[DP]: Au@Ag/PDA-PR-MCS [Anal]: AFP [DM]: H_2_O_2_	[Tran]: amperometric −0.4 V [IC]: (i) AFP conc for 30 min, (ii) [DP] for 1 h [MC]: 5 mM H_2_O_2_, 30 min	[L]: 20fg/mL to 100 ng/mL [LgS] [DL]: 6.7 fg/mL [S]: 16.07 µA/(ng/mL) [SL]: 1 month [CR]: 0.9987	[100]
[DP]: MSN-MB/PDA-anti-PSA [Anal]: PSA [DM]: MB	[Tran]: SWV −0.7 to 0.3 V [IC]: (i) PSA conc for 50 min, 37 °C, (ii) [DP] for 1 h, 37 °C [MC]: 0.1 M HCl, 40 °C, 15 min	[L]: 10 fg/mL to 100 ng/mL [LgS] [DL]: 1.25 fg/mL [S]: 18.84 µA/(ng/mL) [SL]: 30 days [CR]: 0.992	[101]
[DP]: AuPd NCNs-anti-CA 15-3 [Anal]: CA 15-3 [DM]: H_2_O_2_	[Tran]: amperometric 0.2 V [IC]: (i) CA 15-3 conc. drop and dried 4 °C, (ii) [DP] incubation [MC]: 5 mM H_2_O_2_	[L]: 0.001 pg/mL to 100 ng/mL [LgS] [DL]: 0.35 fg/mL [S]: 14.29 µA/(ng/mL) [SL]: 7 days [CR]: 0.9954	[102]
[DP]: Au@Pt DNa/NG/Cu^2+^-anti-CEA [Anal]: CEA [DM]: H_2_O_2_	[Tran]: amperometric −0.4 V [IC]: (i) CEA conc, (ii) [DP] for 40 min [MC]: 5 mM H_2_O_2_	[L]: 0.5 pg/mL to 50 ng/mL [LgS] [DL]: 0.167 pg/mL [S]: 17.9 µA/(ng/mL) [SL]: 3 weeks [CR]: 0.9964	[103]
[DP]: Au@Ag-Cu_2_O/anti-PSA [Anal]: PSA [DM]: 5 mM H_2_O_2_	[Tran]: amperometric −0.4 V [IC]: (i) PSA conc for 1 h, 4 °C, (ii) [DP] [MC]: 5 mM H_2_O_2_	[L]: 0.01 pg/mL to 100 ng/mL [LgS] [DL]: 0.003 pg/mL [S]: 4.98 µA/(ng/mL) [SL]: 16 days [CR]: 0.9998	[104]

Notes: [Ab2]: detection antibody; [Anal]: analyte; [CR]: correlation coefficient; [DL]: detection limit; [DM]: detection molecule; [DP]: detection probe; [IC]: incubation conditions; [L]: linearity; [LgS]: log scale; [LS]: linear scale; [MC]: measurement conditions; [PA]: pulse amplitude; [PP]: pulse period; [PW]: pulse width; [S]: sensitivity; [SL]: shelf life; [Tran]: transducer. 4-CN: 4-chloro-1-naphthol; Aq: anthraquinone 2-carboxylic acid; ASV: anodic stripping voltammetry; ASV: square wave anodic stripping voltammetric measurements; Au AMNPs: Au asymmetric multicomponent nanoparticles; CA 19-9: carbohydrate antigen 19-9; CA15-3: carbohydrate antigen 15-3; CA72-4: carbohydrate antigen 72-4; Cd NCs-Au: Cd nanocubes-gold; CGN: nanocomposite of carbon and gold; Co: Tris(2,2′-bipyridine-4,4′-di-carboxylicacid)cobalt(III)(Co(bpy)33þ, expressed as; CSV: cathodic stripping voltammetry; DPSV: differential pulse stripping voltammetry; Fc: ferrocenecarboxylic acid; MCF: mesoporous carbon form; MCM-41: multifunctional mesoporous silica; M-Pd@Pt: mesoporous core-shell Pd@Pt; M-Pt NPs: mesoporous platinum nanoparticles; MSNs: mesoporous silica nanoparticles; MUC 1: Mucin 1; N-GNRs: N-doped graphene nanoribbons; NP-PtFe: nanoporous PtFe; PB: prussian blue; PBG-Au: poly (brilliant green)-gold; PGN: PtNPs modified graphene nanocomposite; PMCP-Au: poly (*m*-cresol purple)-gold; PPP-Au: poly (*N*-phenyl-*p*-phenylenediamine)-gold; PTBO-Au: poly (toluidine blue o)-gold; SWCNHs: single-walled carbon nanohorns; TB: toluidine blue.
